# SAMD9L acts as an antiviral factor against HIV-1 and primate lentiviruses by restricting viral and cellular translation

**DOI:** 10.1371/journal.pbio.3002696

**Published:** 2024-07-03

**Authors:** Alexandre Legrand, Clara Dahoui, Clément De La Myre Mory, Kodie Noy, Laura Guiguettaz, Margaux Versapuech, Clara Loyer, Margaux Pillon, Mégane Wcislo, Laurent Guéguen, Clarisse Berlioz-Torrent, Andrea Cimarelli, Mathieu Mateo, Francesca Fiorini, Emiliano P. Ricci, Lucie Etienne

**Affiliations:** 1 Centre International de Recherche en Infectiologie (CIRI), Inserm U1111, UCBL1, CNRS UMR 5308, ENS de Lyon, Université de Lyon, Lyon, France; 2 Unité de Biologie des Infections Virales Émergentes, Institut Pasteur, Lyon, Université Paris Cité, Paris, France; 3 Laboratoire de Biologie et Modélisation de la Cellule (LBMC), Université de Lyon, INSERM U1293, CNRS UMR 5239, ENS de Lyon, UCBL1, Lyon, France; 4 Université Paris Cité, CNRS, Inserm, Institut Cochin, INSERM, CNRS, Paris, France; 5 Laboratoire de Biologie et Biométrie Évolutive (LBBE), CNRS UMR 5558, UCBL1, Villeurbanne, France; 6 Retroviruses and structural biochemistry, Molecular Microbiology and Structural Biochemistry (MMSB), IBCP, CNRS UMR 5086, University of Lyon, Lyon, France; The University of Texas Medical Branch at Galveston, UNITED STATES

## Abstract

Sterile alpha motif domain-containing proteins 9 and 9-like (SAMD9/9L) are associated with life-threatening genetic diseases in humans and are restriction factors of poxviruses. Yet, their cellular function and the extent of their antiviral role are poorly known. Here, we found that interferon-stimulated human SAMD9L restricts HIV-1 in the late phases of replication, at the posttranscriptional and prematuration steps, impacting viral translation and, possibly, endosomal trafficking. Surprisingly, the paralog SAMD9 exerted an opposite effect, enhancing HIV-1. More broadly, we showed that SAMD9L restricts primate lentiviruses, but not a gammaretrovirus (MLV), nor 2 RNA viruses (arenavirus MOPV and rhabdovirus VSV). Using structural modeling and mutagenesis of SAMD9L, we identified a conserved Schlafen-like active site necessary for HIV-1 restriction by human and a rodent SAMD9L. By testing a gain-of-function constitutively active variant from patients with SAMD9L-associated autoinflammatory disease, we determined that SAMD9L pathogenic functions also depend on the Schlafen-like active site. Finally, we found that the constitutively active SAMD9L strongly inhibited HIV, MLV, and, to a lesser extent, MOPV. This suggests that the virus-specific effect of SAMD9L may involve its differential activation/sensing and the virus ability to evade from SAMD9L restriction. Overall, our study identifies SAMD9L as an HIV-1 antiviral factor from the cell autonomous immunity and deciphers host determinants underlying the translational repression. This provides novel links and therapeutic avenues against viral infections and genetic diseases.

## Abstract 2

SAMD9 and SAMD9L are known restriction factors of poxviruses, but it is currently unknown if the SAMD9 gene family can restrict other viruses. This study demonstrates that SAMD9L, but not SAMD9, inhibits HIV-1 and other primate lentiviruses through a conserved Schlafen-like active site, thereby disrupting viral translation.

## Introduction

The sterile alpha motif domain-containing proteins 9 and 9-like (*SAMD9* and *SAMD9L)* are paralogous genes that encode large proteins of more than 1,500 amino acids with a complex architecture containing multiple domains. SAMD9 and SAMD9L are involved in several cellular processes such as cell proliferation and protein translation [1–3], apoptosis and stress responses [1], as well as endosomal trafficking [4,5]. Both genes are involved in life-threatening genetic diseases. SAMD9 genetic variants are associated with a multisystem disorder, MIRAGE, and SAMD9L genetic variants are responsible for neurological and hematological disorders, such as ataxia-pancytopenia (ATXPC) or SAMD9L-associated autoinflammatory disease (SAAD) [2,6–8]. These syndromes reflect gain-of-function (G-o-F) phenotypes caused by missense mutations or truncated forms. The underlying causes appear a consequence of accrued translational repression by SAMD9L G-o-F variants, although other mechanisms may also be involved [5,9,10].

SAMD9 and SAMD9L are also restriction factors of poxviruses, acting through virus translational repression [11–13]. Double-stranded nucleic acid (dsNA) binding appears an important determinant of antipoxviral and antiproliferative activity of SAMD9/9L [14]. Yet, mammalian poxviruses encode species-specific antagonists from the C7K superfamily, which are necessary for viral replication and pathogenesis [11–13]. Whether the SAMD9 gene family can restrict other viruses is currently unknown.

The human immunodeficiency virus type 1 (HIV-1) is responsible for acquired immunodeficiency syndrome (AIDS) and remains a major public health concern with 38 million people living with the virus in 2021 (UNAIDS). The identification and characterization of cellular proteins that naturally inhibit HIV and related viruses have been key to understanding HIV infection and mammalian antiviral innate immunity [15–17]. Furthermore, several factors at the interface with HIV are also involved in major cellular dysfunctions, such as the HIV restriction factor SAMHD1 also involved in Aicardi–Goutières syndrome (AGS) [18]. Overall, unveiling host factors that impact HIV at different steps of replication and that are possibly involved in cellular dysfunctions helps to design new drug targets against viral pathogens and genetic diseases.

Here, by combining protein structure and genetic analyses, with molecular virology and functional characterization, we show that the interferon-induced SAMD9L, and not SAMD9, specifically inhibits HIV and other lentiviruses, through a Schlafen-like (SLFN) ribonuclease site driving SAMD9L cellular and antiviral functions. Our study, therefore, identifies a novel ISG-encoded protein with antiviral functions against HIV. It also provides evidence of functional differences between SAMD9 and SAMD9L with regard to viruses and extends SAMD9L antiviral defense beyond poxviral DNA viruses.

## Results

### SAMD9L, and not SAMD9, acts as an antiviral factor against HIV-1 and primate lentiviruses

**Fig 1:**
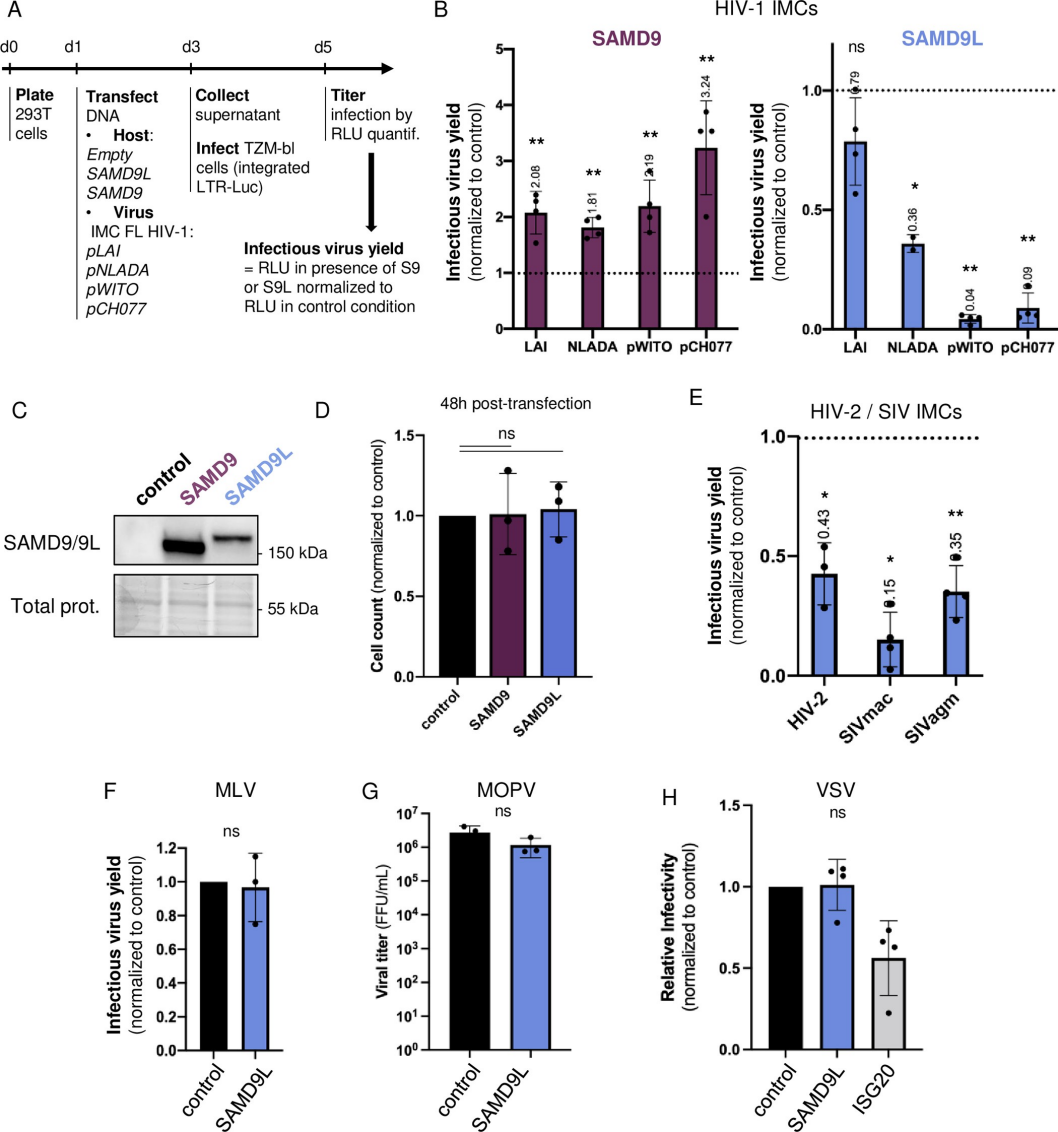
Ectopically expressed SAMD9L, but not SAMD9, specifically restricts HIV-1 and lentiviruses. (**A**) Experimental setup for the ectopically expressed SAMD9/9L experiments. Host and virus DNA, and cells as described in Methods. (**B**) Relative infectious virus yields of 4 HIV-1 strains in the context of SAMD9 or SAMD9L expression (1,500 ng DNA), normalized to the control in the absence of SAMD9/9L (empty plasmid with the corresponding viral strain). Infectious virus yield was measured as shown in panel (**A**). Corresponding western blot analyses showing SAMD9/9L expression are shown in S1A Fig. (C) Western blot analyses of ectopically expressed SAMD9 (anti-Flag) and SAMD9L (anti-SAMD9L) expression. “Total prot.” corresponds to the total proteins’ expression and serves as control (see Methods). It further shows that SAMD9/9L ectopic expressions do not impact total cellular protein levels in these experimental settings (S1B Fig). (**D**) Relative cell count at 48 h posttransfection of control, SAMD9 or SAMD9L plasmid (1,500 ng) in 293T cells. (**E**) Same as in 1A and 1B with HIV-2, SIVmac, and SIVagm IMCs. (**F**) Infectious virus yield of retroviral MLV:GFP particles produced with or without SAMD9L, measured by the % of GFP positive cells in 293T target cells (normalized to the control with an empty plasmid; S2D and S2E Fig). (**G**) MOPV viral titers retrieved from control cells or cells overexpressing SAMD9L. Titers were measured as FFUs. (**H**) 293T cells transfected with an empty plasmid, SAMD9L, or ISG20 (as positive antiviral control) were infected 24 h later with VSV:GFP replicative particles, and supernatant was collected 16 hpi. The viral titer was measured in new cells by the % of GFP-positive cells. Results are expressed as normalized to the empty control. All panels, statistics versus the corresponding control condition: **, *p*-value < 0.01; *, *p*-value < 0.05; ns, *p*-value > 0.05. The data and the raw images underlying this Figure can be found in S1 Data and S1 Raw Images. FFU, focal forming unit; IMC FL HIV-1, infectious molecular clone full-length HIV-1; RLU, relative light unit; S9, SAMD9; S9L, SAMD9L.

SAMD9L has been recently retrieved as a potential HIV modulator in a genome-wide CRISPR-based screen [19], but its potential anti-HIV function was not investigated. To determine if SAMD9 and SAMD9L restrict HIV-1, we first tested the capacity of the ectopically expressed proteins to impact the infectious virus yield of 4 full-length infectious molecular clones (IMCs) of HIV-1 viruses. We tested 2 HIV-1 laboratory-adapted strains, LAI and NLADA, which use CXCR4 (X4) and CCR5 (R5) as coreceptors for entry, respectively. We also tested 2 HIV-1 transmitted/founder (T/F) primary strains, the R5/X4-tropic pCH077 and the R5-tropic pWITO, which correspond to initial natural viral variants responsible for a productive infection in HIV-1-infected people [20,21]. To do so, we cotransfected 293T cells with or without a SAMD9/9L-encoding plasmid and individual proviral clones (IMCs), and we titered the HIV-1 infectious yield in the supernatant by infecting the TZM-bl reporter cell line, which encodes for luciferase downstream of an LTR promoter (Fig 1A). Under these conditions, we found that SAMD9L inhibited HIV-1 infectious virus yield, while SAMD9 exerted an opposite effect, enhancing HIV-1 (Figs 1B, 1C, and S1A). Of note, in these conditions, exogenous SAMD9/9L by themselves did not impact cell counts or total steady-state protein expression levels (Figs 1C, 1D, and S1B). Interestingly, SAMD9L strongly restricted the 2 HIV-1 T/F strains—with 1 log restriction in infectious virus yields (Fig 1B). Experiments with decreasing/increasing amounts of HIV-1 IMCs in the producer cells confirmed the HIV-1 inhibition by SAMD9L. They also suggest possible HIV-1 strain-specific effect, with a strong inhibition of HIV-1 pWITO as compared to HIV-1 LAI, which seemed, at least partly, independent of the viral doses and infectious yields (S2A and S2B Fig). SAMD9L restriction also appeared independent of the coreceptor use. Lastly, the inhibition of HIV-1 was dependent on the dose of SAMD9L (S2C Fig). Because of the strong antiviral effect of SAMD9L on primary HIV-1 isolates, we focused the study on this immune defense protein.

We next tested SAMD9L ability to impact the infectious virus yield of several human and simian lentiviruses (simian immunodeficiency viruses (SIVs)) beyond the pandemic HIV-1. We found that SAMD9L could also restrict the distantly related HIV type 2, HIV-2 (Fig 1E). Furthermore, SAMD9L also strongly restricted the infectious yield of IMCs from simian lentiviruses, SIVagm.Tan1 from African green monkey tantalus, and SIVmac from rhesus macaque (Fig 1E), indicating that SAMD9L is an antiviral factor of primate lentiviruses.

Finally, to determine whether SAMD9L broadly affected more distantly related viruses, we tested its activity against the gammaretrovirus MLV (murine leukemia virus) in addition to 2 RNA viruses: mopeia virus (MOPV) from the *Arenaviridae* family and vesicular stomatitis virus (VSV) from the *Rhabdoviridae* family (see Methods for experimental setup). In the context of SAMD9L exogenous expression, we found no significant changes in viral titers (Figs 1F–1H, S2D, and S2E; for VSV, we used the antiviral ISG20 as positive control [22]). A dose of MLV-coding DNA plasmids (from 400 ng to 1,500 ng) in the context of SAMD9L confirmed the absence of restriction in the infectious yield (S2E Fig). This showed a lentivirus-specific antiviral effect of SAMD9L, regarding the other retrovirus and RNA viruses tested in our conditions.

### SAMD9L restricts HIV-1 late steps, after viral transcription and before maturation

**Fig 2:**
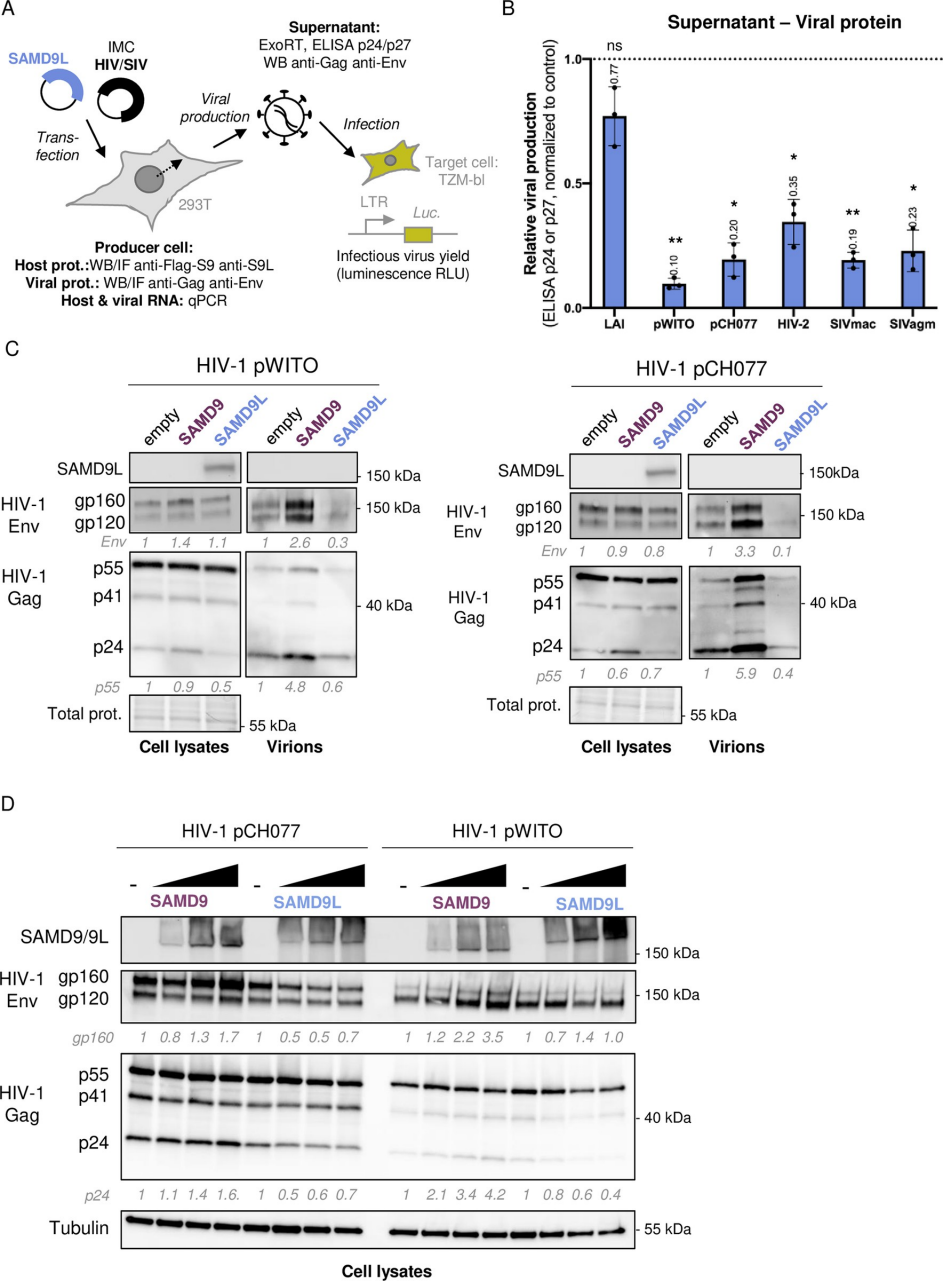
SAMD9L restricts lentiviral protein production. (**A**) Experimental setup. Setup similar to Fig 1A with detailed assays to investigate the viral restriction step. (**B**) Relative viral production of diverse primate lentiviruses, as measured by ELISA p24 (for HIV-1 strains) or p27 (for HIV-2 and SIVs) in the supernatant of producer cells with SAMD9L, normalized to the control. (**C**) Western blot analyses of SAMD9L and HIV-1 Env and Gag proteins in the viral producer cells and in the supernatant for 2 HIV-1 T/F strains. Quantification is provided for Env and Gag p55 protein expression, normalized to the total proteins in the cell fraction, and expressed as fold difference compared to the control (normalized to 1). (**D**) Similar to C in the context of increasing amount of SAMD9 and SAMD9L. Quantification is provided for gp160 and Gag p24 protein expressions normalized to Tubulin, and expressed as fold-difference compared to the control (normalized to 1). Statistics: **, *p*-value < 0.01; *, *p*-value < 0.05; ns, *p*-value > 0.05. The data and the raw images underlying this Figure can be found in S1 Data and S1 Raw Images. HIV-1, human immunodeficiency virus type 1; SAMD9, sterile alpha motif domain-containing protein 9; SAMD9L, sterile alpha motif domain-containing protein 9-like; SIV, simian immunodeficiency virus; T/F, transmitted/founder.

To identify the lentiviral replication step affected by SAMD9L, we analyzed the levels of proteins secreted in the supernatant, by quantification of Gag p24 or p27 ELISA, and we assessed for viral and host proteins in the viral supernatant and in the producer cells, by western blot analyses of HIV-1 structural Env and Gag proteins and SAMD9L (Fig 2A). We further kept the SAMD9 condition, as a control of the SAMD9L-specific effect in our experimental settings.

Under these conditions, SAMD9L significantly decreased the amount of viral Gag (p24 or p27 ELISA) from HIVs and SIVs in the viral supernatant, which correlated with the virus infectious yields (Figs 2B and S3A). Western blot of the purified HIV-1 virions in the supernatant fraction further showed that SAMD9L induced a marked decrease of both Gag and Env viral protein expressions, while SAMD9 had an enhancing effect (Fig 2C). Intracellular expression of the viral proteins was also affected, showing that SAMD9L restricts lentiviral protein expressions in the producer cells (Figs 2C, 2D, S3B, S3C, and S4). Because the protein inhibition seemed much stronger in the viral supernatant fraction as compared to the cellular fraction and, in particular, for HIV-1 Env (Figs 2C and S4), it is possible that SAMD9L also affects viral protein trafficking and Env incorporation.

**Fig 3:**
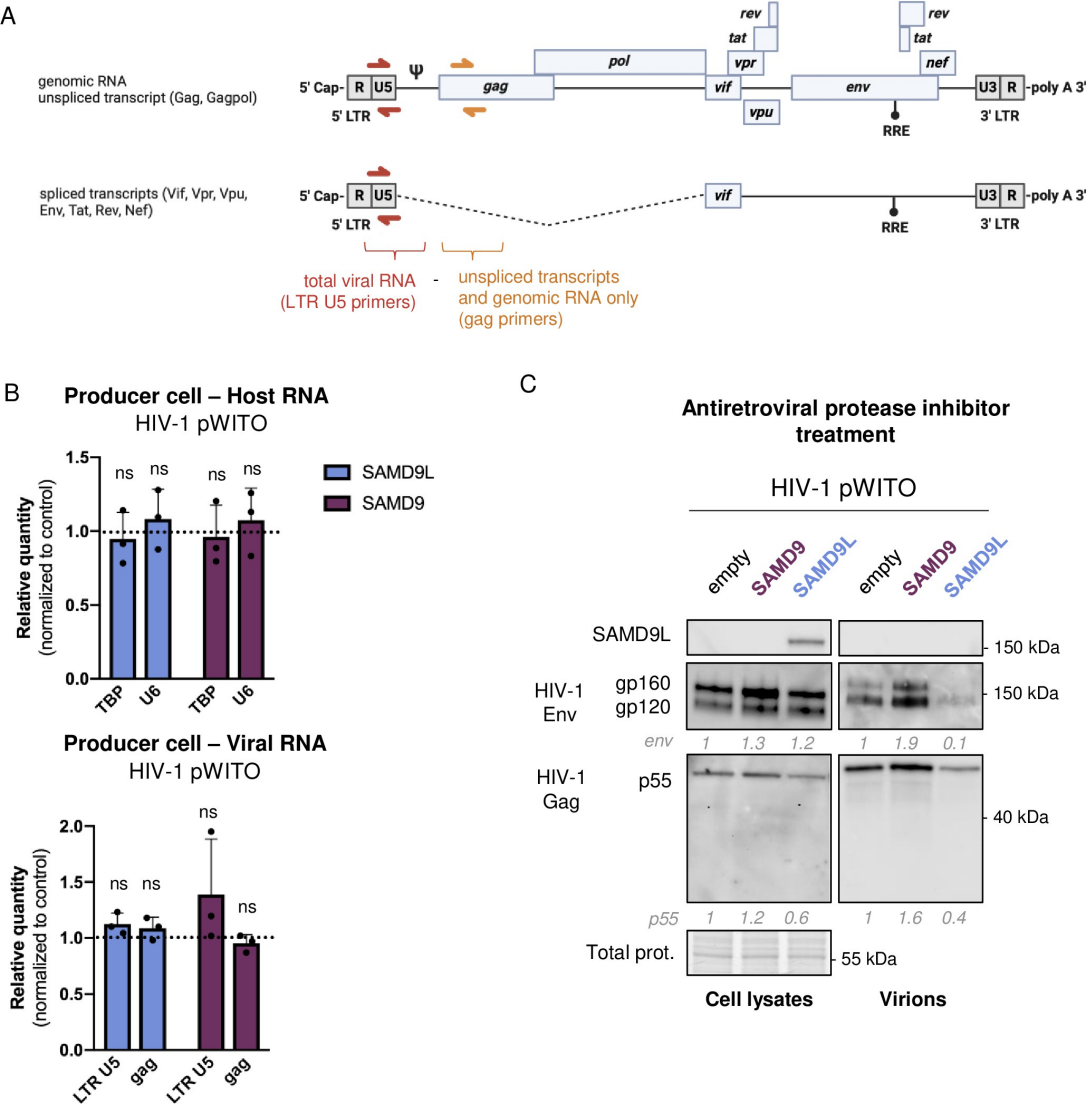
SAMD9L does not impair HIV-1 RNA levels, nor HIV-1 protein maturation. (**A**) qPCR experimental setup: Regions and types of HIV-1 RNAs targeted by the qPCR primers. (**B**) Relative RNA quantity in the HIV-1 producer cells for total viral RNA (LTR U5) or unspliced transcripts and gRNA (gag) in +/− SAMD9 or SAMD9L conditions. TBP and U6 are similarly assessed as cellular controls. (**C**) Same as Fig 2C under antiretroviral protease inhibitor treatment. The data and the raw images underlying this Figure can be found in S1 Data and S1 Raw Images. gRNA, genomic RNA; HIV-1, human immunodeficiency virus type 1; SAMD9, sterile alpha motif domain-containing protein 9; SAMD9L, sterile alpha motif domain-containing protein 9-like; TBP, TATA-binding protein.

To determine whether SAMD9L modulated viral transcription or the quantity of the newly synthesized HIV-1 RNAs, we performed quantitative reverse transcription PCRs (RT-qPCRs) targeting 2 HIV-1 regions, gag and LTR U5, of the HIV-1 pWITO transcripts in the producer cells (Fig 3A). In this experimental design, total HIV-1 RNA, which includes viral genomic RNA (gRNA), spliced and unspliced mRNAs, was quantified using the LTR U5 primers, while the HIV-1 gRNA and the unspliced HIV-1 mRNAs were measured using the *gag* primers. We also used 2 host cell RNA controls: the nuclear RNA U6 and the TATA-binding protein (TBP). We found that SAMD9 and SAMD9L did not modulate any HIV-1 RNA species or types, nor the cellular U6 and TBP RNAs (Fig 3B). This shows that SAMD9 and SAMD9L do not impact the amount nor the splicing of HIV-1 RNAs, implying a posttranscriptional effect.

Because of potential differences in the expression levels between viral Gag products (Figs 2C, 2D, S3B, and S4), which could result from an impairment of HIV-1 Gag maturation, we explored SAMD9L effect on HIV-1 upon a treatment with antiretroviral protease inhibitors, Saquinavir and Indinavir. We found that the SAMD9L-induced defect of Gag p55 production and expression, in the cell and supernatant, were clearly visible upon antiprotease treatment, showing a prematuration effect of SAMD9L (Fig 3C).

Lastly, we tested whether SAMD9L could also impact the early phases of HIV-1 and SIVmac replication. We used single-round nonreplication-competent viruses (HIV-1 and SIVmac viral-like particles pseudotyped with VSVg and encoding for a firefly luciferase reporter gene in the transfer plasmid, HIV-1:Luc and SIVmac:Luc, respectively) to infect target cells overexpressing SAMD9L (S5A Fig). We found that target cells overexpressing SAMD9L had similar levels of incoming single-round lentiviral infection compared to control cells (S5 Fig), showing that SAMD9L does not impact the early phases of lentiviral replication.

Therefore, we show that SAMD9L restricts HIV-1 and other lentiviruses’ replication by affecting their late phases, after the transcription and splicing, and before the maturation steps.

### SAMD9L is an ISG that restricts HIV-1 replication in IFN-I-stimulated cells

**Fig 4:**
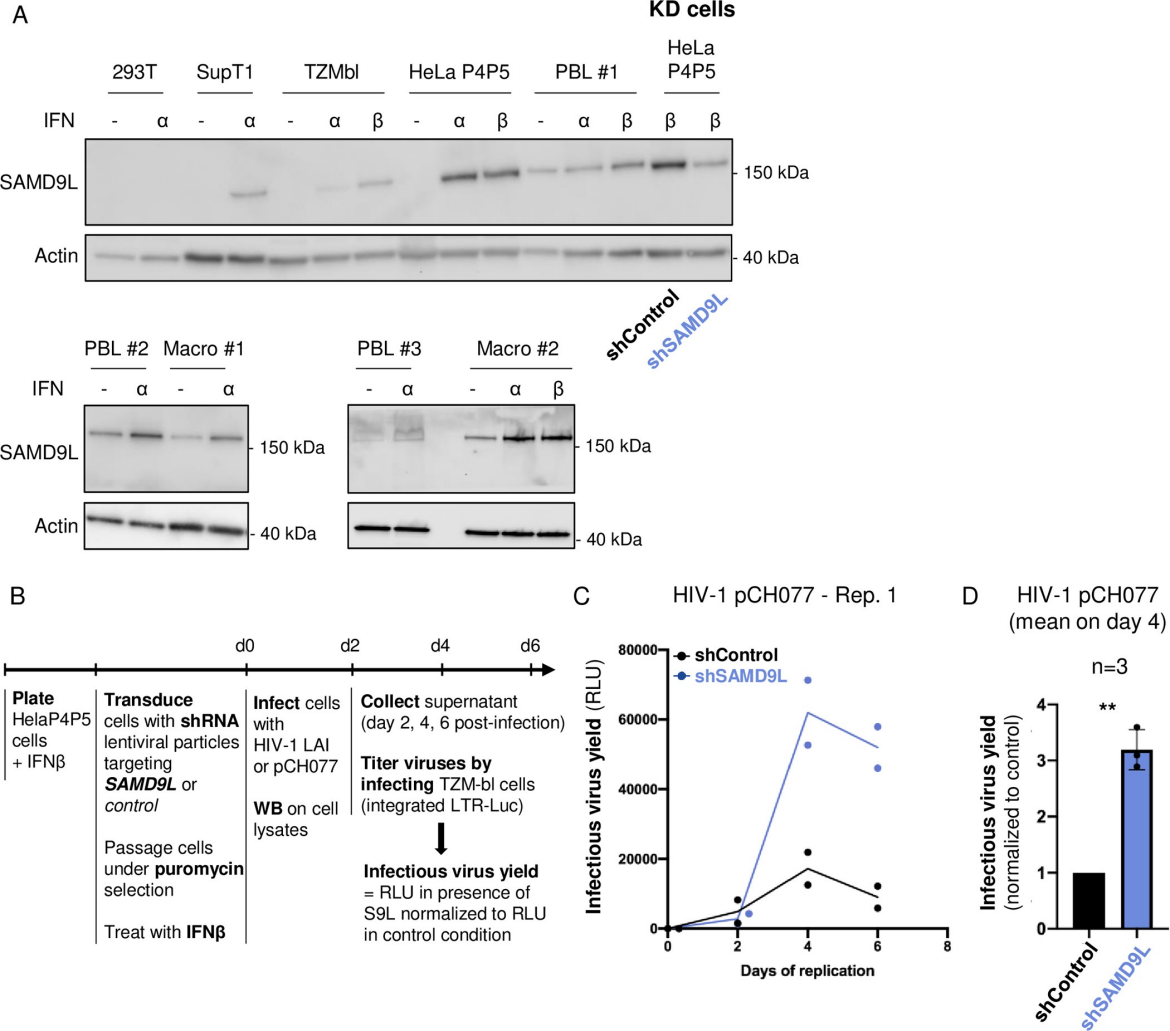
SAMD9L is an ISG that endogenously restricts HIV-1 replication. (**A**) Western blot analyses of endogenous SAMD9L expression under basal, IFN⍺, or IFNβ stimulation in various cells: 293T cells, immortalized T cell line SupT1, immortalized fibroblastic cell lines (TZM-bl, HeLaP4P5), PBLs from 3 donors, and primary macrophages from 2 donors. The last line of the first blot shows the condition of KD HelaP4P5 cells stimulated with IFNβ and transduced with either shControl (shCD241) or shSAMD9L (performed at day 0 of the viral replication experiment). (**B**) Experimental setup for the KD and viral replication experiments. (**C**) HIV-1 T/F pCH077 replication assay in shControl or shSAMD9L HeLaP4P5 +IFNβ over 6 days of replication. Titration was performed using the TZM-bl assay. Y-axis represents the raw RLU results for 1 experiment with the technical replication duplicates for each condition. Three independent biological replicates were performed for HIV-1 pCH077 and pCH077:VSVg, and 2 for LAI (S6 Fig). (**D**) The mean of 3 independent experiments is shown for HIV-1 pCH077 replication in shSAMD9L conditions normalized to shControl. **, *p*-value < 0.01. The data and the raw images underlying this Figure can be found in S1 Data and S1 Raw Images. HIV-1, human immunodeficiency virus type 1; IFN, interferon; ISG, interferon-stimulated gene; KD, knock-down; PBL, primary blood lymphocyte; RLU, relative luminescence unit; SAMD9L, sterile alpha motif domain-containing protein 9-like; T/F, transmitted/founder.

We next determined SAMD9L endogenous expression in basal condition and upon type I interferon (IFNα and IFNβ) stimulation, by western blot analyses of diverse cell lines in our experimental settings and in natural HIV target cells. We found that endogenous SAMD9L expression was undetectable in all immortalized cell lines at basal condition (Fig 4A). Upon IFN-I stimulation, SAMD9L expression was detectable in SupT1 T cell line, TZM-bl and HelaP4P5 cells (a HeLa cell line engineered to express CD4^hi^CCR5^hi^CXCR4^hi^ to serve as HIV target cells) (Fig 4A). Furthermore, endogenous SAMD9L was expressed in primary macrophages and in primary blood lymphocytes (PBLs), and its expression was also dependent on type I IFN (Fig 4A). These results confirm that SAMD9L is an interferon-stimulated gene (ISG) [7] with limited expression at basal condition.

To determine the functional relevance of endogenous SAMD9L on HIV-1 replication, we silenced SAMD9L expression, by shRNA gene transduction (shSAMD9L and shControl, as in [23]) in HIV-1-permissive cells under IFN-I stimulation. We used the HeLaP4P5 cells under IFNβ stimulation to perform the HIV-1 replications, as the levels of SAMD9L expression and silencing in these cells were validated and comparable to primary lymphocytes and macrophages (Fig 4A showing SAMD9L expression in shControl and shSAMD9L cells). Knock-down (KD) cells were infected in technical duplicates with viral titers equivalent to 10 ng of p24 Gag of infectious HIV-1 T/F pCH077, HIV-1 LAI, or HIV-1 T/F pCH077:VSVg (i.e., the VSVg-dependent entry is only involved in the first round of replication). We monitored viral replication over a 6-day period, with supernatant being collected every 2 days and titered using TZM-bl reporter cells (Fig 4B). The complete experiment was performed in 2 to 3 independent biological replicates (Figs 4 and S6). We found that HIV-1 infectious titers, over a 6-day replication curve, were higher in the shSAMD9L cells compared to the shControl cells, with a significant 3-fold increase for HIV-1 pCH077 at day 4 (Figs 4D and S6). This suggests that endogenous IFN-stimulated SAMD9L inhibits HIV-1 replication.

### HIV and lentivirus restriction by SAMD9L depends on E198/D243 in a SLFN-like active site

**Fig 5:**
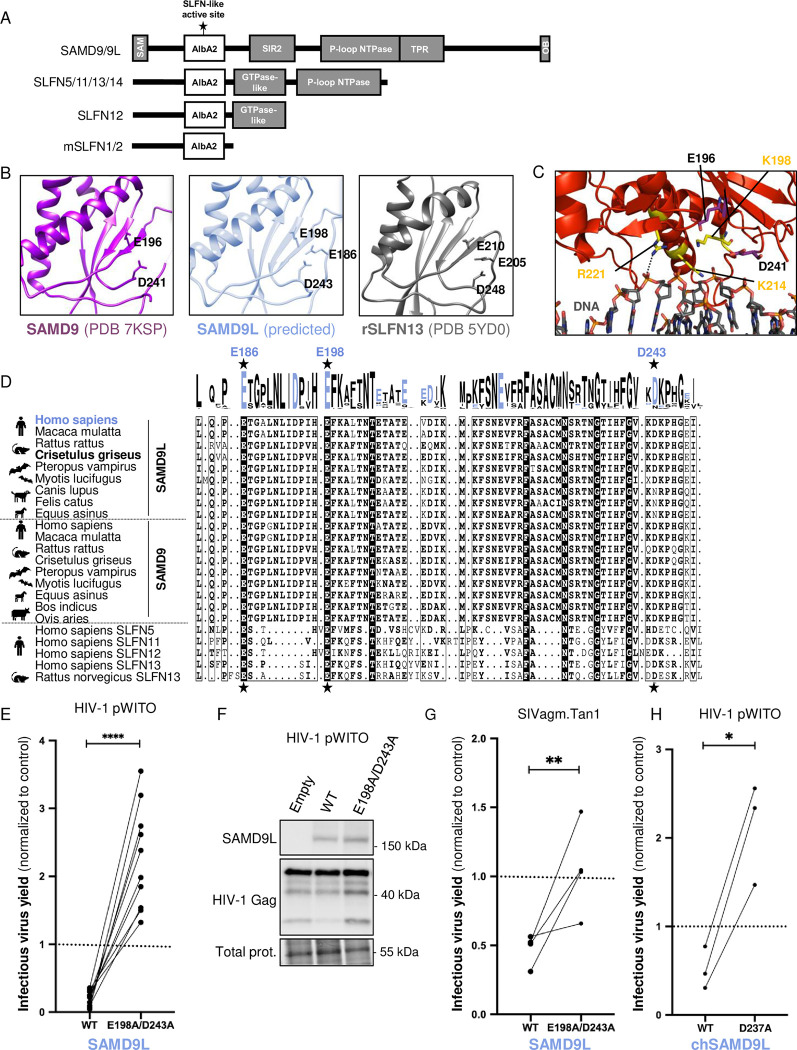
SAMD9L restriction of lentiviruses is governed by residues E198/D243 in a SLFN-like box active site, conserved in mammals. (**A**) Representation of the protein domains in SAMD9/9L and SLFNs with the identification of the putative active site in a SLFN-like box. (**B**) Structure of the SLFN-like box in SAMD9, SAMD9L, and rSLFN13. Atoms of the residues forming the active site are represented with sticks and their coordinates labeled in black. SAMD9 and rSLFN13 are from solved crystal structures (PDB 7KSP and 5YD0, respectively), while SAMD9L is from AlphaFold prediction. (**C**) SAMD9 crystal structure in complex with DNA (PDB 7KSP). Atoms of the residues forming the active site are represented with purple sticks (labels in black), while those involved in DNA binding are in yellow. (**D**) Amino acid sequence alignment of the region bearing the SLFN-like box in SAMD9/9L and SLFNs from various mammalian species. The triad of residues forming the active site is labeled with black stars with coordinates from human SAMD9L above in blue. In the alignment: Residues with black font are strictly conserved among the sequences; dots correspond to alignment gaps; bold residues are the one matching with the consensus. The logo plot is from WebLogo with the acidic residues (Asp, D and Glu, E) in blue. (**E**) Relative infectious virus yield of HIV-1 pWITO in empty, SAMD9L or SAMD9L-E198A/D243A conditions, following Fig 1A experimental design. (**F**) Western blot analysis in the corresponding producer cells. Loading control is from total proteins. (**G**) Similar to panel (**E**) with SIVagm.Tan1. (**H**) Similar with Chinese hamster chSAMD9L WT and D237A on HIV-1 pWITO infectious virus yield. Statistics: ****, *p*-value < 0.0001; **, *p*-value < 0.01; *, *p*-value < 0.05. The data and the raw images underlying this Figure can be found in S1 Data and S1 Raw Images. HIV-1, human immunodeficiency virus type 1; mSLFN1/2, mouse SLFNs; rSLFN13, rat SLFN13; SAMD9, sterile alpha motif domain-containing protein 9; SAMD9L, sterile alpha motif domain-containing protein 9-like; SLFN, Schlafen; WT, wild-type.

SAMD9 and SAMD9L are large proteins predicted to have a similar and complex domain architecture (Fig 5A) [24]. The recently solved crystal structure of the SAMD9 156–385 amino acid region showed it can bind DNA [14]. This region corresponds to the AlbA2 domain, which typically binds DNA and/or RNA and possesses multimerization properties [24,25]. Before the SAMD9(156–385) structure was available (S7A Fig), we had analyzed SAMD9L with the protein homology and structure prediction tool HHpred and found a homology with members of the Schlafen (SLFN) gene family (S7B Fig). This homology localized into their respective AlbA2, which is present in all SLFNs and forms the C-lobe of their N-terminal domain, also known as the SLFN-box [26] (Fig 5A). SLFN genes are involved in multiple cellular processes, including development, cancer, and antiviral immunity [26]. The SLFN-box presents nucleic acid binding properties, as well as endoribonuclease activity in several SLFNs, and the enzymatic active site is dependent on 3 conserved acidic amino acids (2 Glu (E) and 1 Asp (D)) [26,27]. Interestingly, SLFN11, SLFN12, and SLFN13 restrict HIV-1 replication by inhibiting translation, and this enzymatic active site is necessary for this function [27–31]. We therefore compared the structures of the SAMD9-AlbA2 domain, the AlphaFold-predicted SAMD9L, and the SLFN-box of SLFN5, SLFN12, and rSLFN13. We found strong structural homology and conservation of the active site triad in SAMD9/9L: E184/E196/D241 in SAMD9 and E186/E198/D243 in SAMD9L (Figs 5B, 5C, S7C, and S7D). Although the flanking regions in the 2D sequences were different between SLFNs and SAMD9/9Ls, a protein sequence alignment showed a strong conservation of these 3 residues in SAMD9/9L mammalian sequences (Fig 5D). We therefore suspected their key role in SAMD9L antiviral function against lentiviruses.

To test this, we mutated the active site triad at 2 residues generating the SAMD9L-E198A/D243A mutant plasmid, and we assessed its effect on HIV-1 pWITO and SIVagm.Tan1. We found that the HIV-restriction ability of SAMD9L was totally abolished by the E198A/D243A mutations (Fig 5E). In accordance, the expression of HIV-1 structural proteins in the cell was higher in the presence of SAMD9L-E198A/D243A compared to wild-type (WT) SAMD9L (Fig 5F). Furthermore, SAMD9L antiviral effect against SIVagm.Tan1 was also dependent on the E198/D243 site (Fig 5G).

To determine if the antiviral function and the motif were also conserved and important in another mammalian SAMD9L ortholog, we tested the Chinese hamster SAMD9L (chSAMD9L) [32] and its respective mutant in the predicted active site, chSAMD9L-D237A. We found that chSAMD9L also restricted HIV-1 pWITO and that chSAMD9L-D237A lost its antilentiviral capacity (Fig 5H), showing that this catalytic site is also necessary in chSAMD9L for lentiviral restriction.

Altogether, we identified and showed that the antilentiviral activity of SAMD9L is dependent on the identified SLFN-like active site.

### Mutating the SLFN-like active site also relieves the translational inhibition of a pathogenic gain-of-function SAMD9L

**Fig 6:**
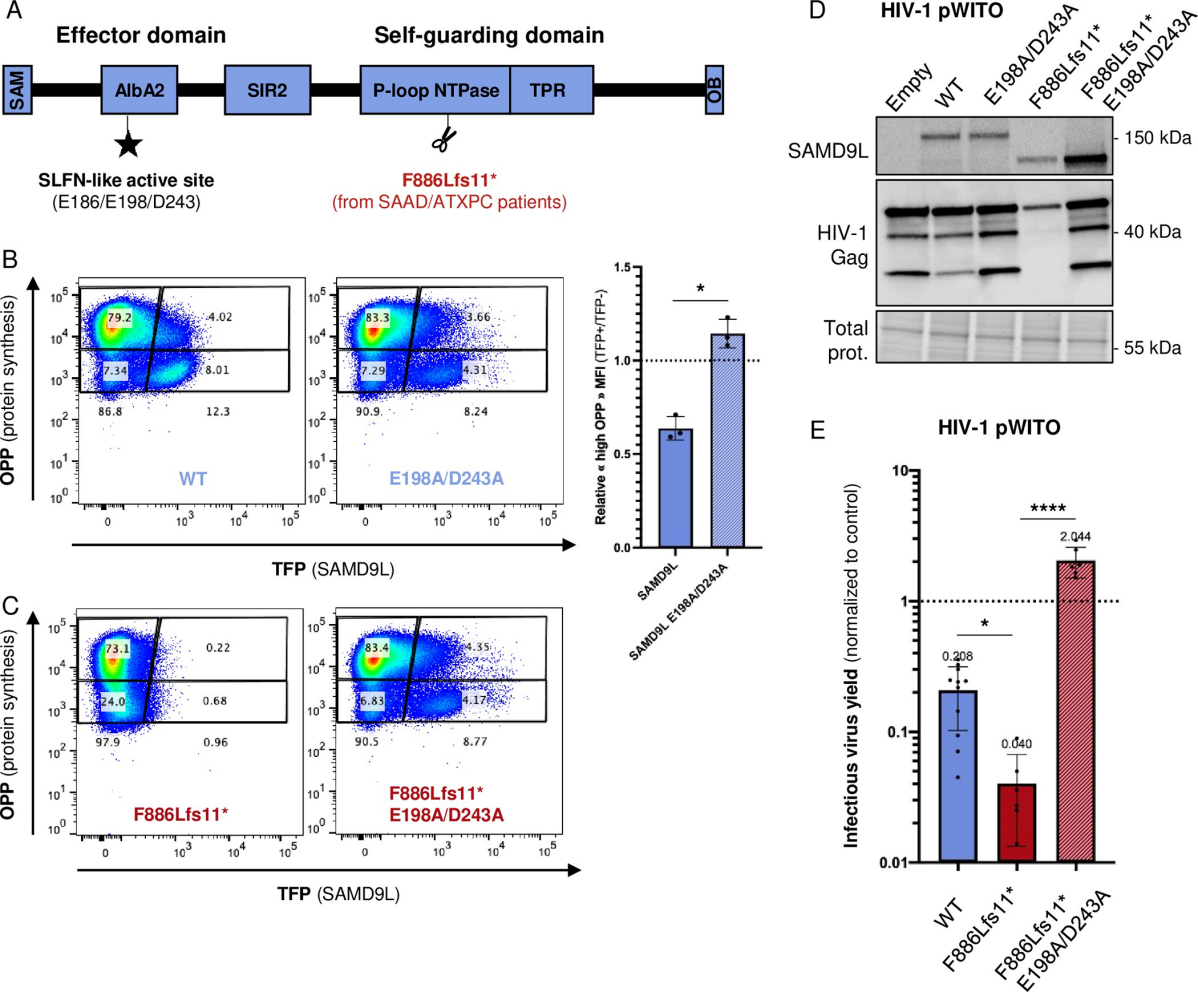
The cellular translational repression of SAMD9L and G-o-F variant is alleviated by the E198A/D243A mutations in the SLFN-like box. (**A**) Representation of the protein domains in SAMD9L highlighting the truncation of 1 patient variant, SAMD9L-F886Lfs11* [2], and the SLFN-like box active site identified in this study. (**B**) Representative flow cytometry density plots of OPP incorporation relative to the expression of either TFP-SAMD9L WT or WT E198A/D243A. Plots are divided into 4 polygons representing TFP+ cells (right), TFP− cells (left), high OPP cells (up), and low OPP (down). Corresponding cell percentages are labeled into corresponding polygons and sums of TFP+ and TFP− cells are labeled below. On right, quantification from 3 independent biological replicates. For each replicate, the relative “high OPP” MFI was calculated using the MFI of the “high OPP” TFP+ polygon normalized to the MFI of the “high OPP” TFP− polygon. (**C**) Similarly, effect of F886Lfs11* and F886Lfs11* E198A/D243A. Of note and as previously reported, SAMD9L-F886Lfs11* also represses its own protein synthesis. (**D**, **E**) Effect of SAMD9L patient variant and E198/D243 mutants on HIV-1 protein expression (**D**) and relative infectious virus yield (**E**). Experimental conditions as in Fig 2A. Statistics: ****, *p*-value < 0.0001; *, *p*-value < 0.05. The data, the raw images, and the FCS files and gating strategy underlying this Figure can be found in S1 Data, S1 Raw Images, and S2 Data, respectively. G-o-F, gain-of-function; HIV-1, human immunodeficiency virus type 1; MFI, median fluorescence intensity; OPP, O-propargyl-puromycin; SAMD9L, sterile alpha motif domain-containing protein 9-like; SLFN, Schlafen; WT, wild-type.

Germline G-o-F mutations in SAMD9L are responsible for diverse multisystem disorders, including myeloid malignancies and systemic autoinflammatory diseases in humans [2,6–8]. These mutations are predominantly localized in its C-terminal half-part, mostly within the putative P-loop NTPase domain [8]. One such variant from SAAD/ATXPC patients is SAMD9L-F886Lfs*11, a germline frameshift mutant that generates a C-terminal truncated protein [2] (Fig 6A). It is associated with a G-o-F constitutively active phenotype, dramatically suppressing cellular protein synthesis [1–3]. The basic residues (K or R) in the SAMD9L AlbA2, which are involved in dsNA binding, are necessary for this translation inhibition [14]. To determine the importance of the SLFN-like acidic E198/D243 residues on cellular translation inhibition, we performed a Click-iT Plus O-propargyl-puromycin (OPP) Synthesis Assay, by overexpressing in 293T cells SAMD9L WT, SAMD9L-F886Lfs*11 (i.e., SAMD9L truncation based on the patient’s natural variant), or their corresponding mutants E198A/D243A, and by measuring OPP incorporation in nascent cellular proteins by flow cytometry. We showed that the mild translational repression by SAMD9L WT and the strong inhibition exerted by the SAMD9L-F886Lfs*11 were strictly dependent on E198/D243 (Fig 6B and 6C). Therefore, E198/D243 of the SLFN-like box active site in SAMD9L are essential and drive cellular protein synthesis repression.

Furthermore, we tested how SAMD9L-F886Lfs*11 impacted lentiviral replication and found that it strongly inhibited HIV-1 protein expression and infectious virus yield, increasing the antiviral effect by 5-fold compared to SAMD9L WT (Fig 6D and 6E). This viral restriction was also fully abolished by the E198A/D243A mutations. Therefore, the pathogenic G-o-F SAMD9L-F886Lfs*11 exerts an increased antilentiviral activity, and, importantly, the exacerbated shutdown of both cellular and lentiviral protein synthesis can be relieved by mutating the SLFN-like active site.

### The gain-of-function SAMD9L can restrict MLV and, to a lesser extent, MOPV

As SAMD9L did not impact other RNA viruses tested in this study, we wondered whether the G-o-F constitutively active form of SAMD9L could restrict their replication. We therefore assessed the effect of SAMD9L-F886Lfs*11 on the gammaretrovirus MLV and on the RNA arenavirus MOPV.

**Fig 7:**
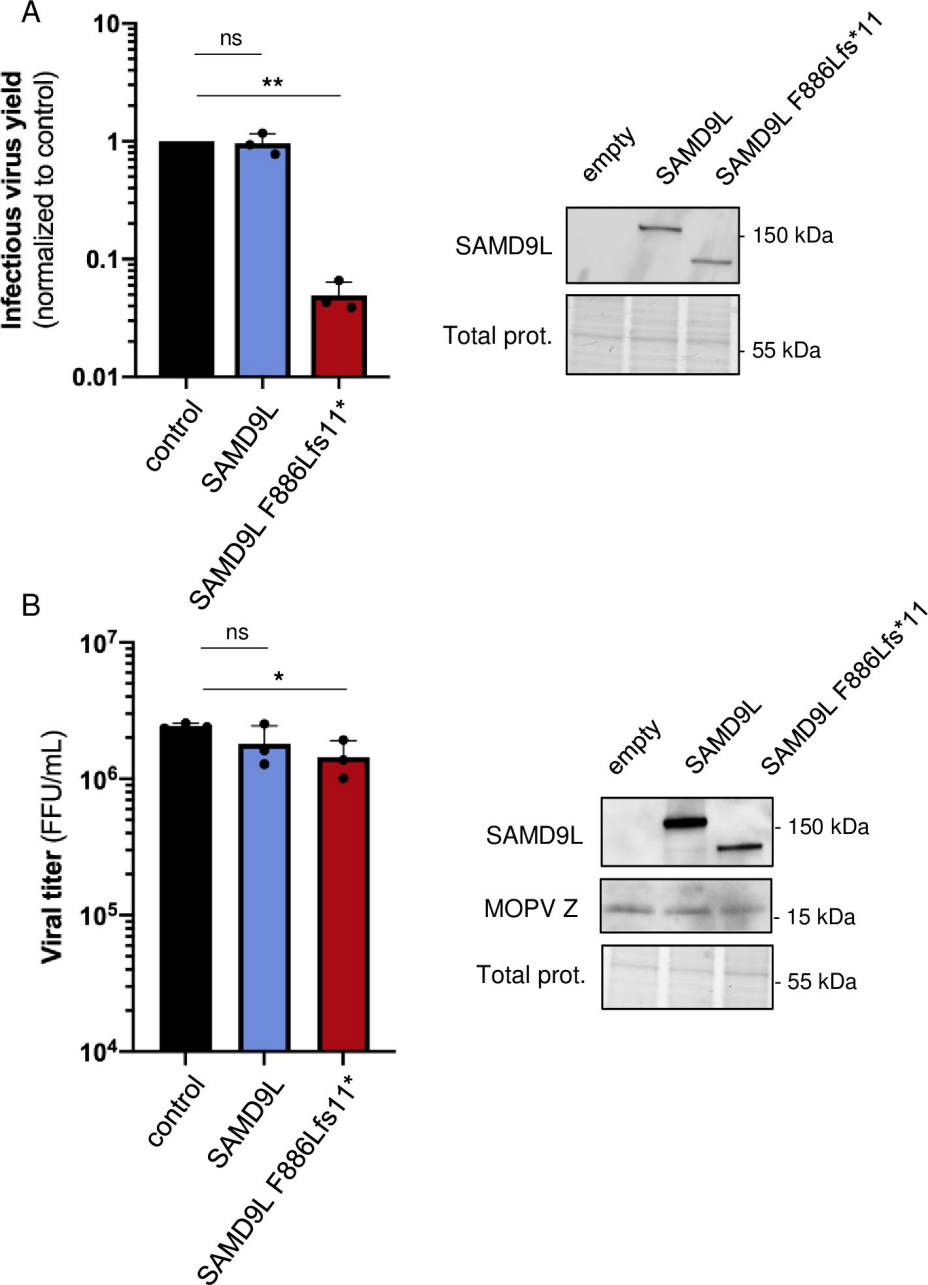
MLV and MOPV are strongly and mildly affected by the translational repression of G-o-F SAMD9L, respectively. (**A**) Infectious virus yield of retroviral MLV:GFP particles produced with or without SAMD9L, measured by the % of GFP-positive cells in 293T target cells (normalized to the control). Right, western blot analyses of SAMD9L in the MLV producer cells. (**B**) MOPV viral titers retrieved from control cells or cells overexpressing SAMD9L. Titers were measured as FFUs. Right, western blot analyses of SAMD9L and MOPV Z proteins for MOPV-infected cells overexpressing SAMD9L. Statistics: **, *p*-value < 0.01; *, *p*-value < 0.05; ns, *p*-value > 0.05. The data and the raw images underlying this Figure can be found in S1 Data and S1 Raw Images. FFU, focal forming unit; G-o-F, gain-of-function; MLV, murine leukemia virus; MOPV, mopeia virus; SAMD9L, sterile alpha motif domain-containing protein 9-like.

We found that, although MLV infectious virus yield was not affected by WT SAMD9L, it was strongly restricted (20-fold restriction) by the G-o-F SAMD9L (Fig 7A). This indicates that MLV is not intrinsically resistant to SAMD9L translational shutdown activity. The main difference between WT and the G-o-F SAMD9L is the exacerbated and constitutive activity, likely due to a defect in autoinhibition, which bypass the sensing and activation steps. Therefore, the results suggest that, once active, SAMD9L can strongly block the late phases of MLV replication.

We also found that SAMD9L-F886Lfs*11, as opposed to WT SAMD9L, could restrict MOPV replication titers (Fig 7B). Yet, the restriction was only of 2-fold comparing to the control condition (1.4 × 10^6^ FFU/ml versus 2.4 × 10^6^ FFU/ml; Fig 7B). Western blot analyses allowed us to show that MOPV replication did not affect steady-state SAMD9L WT and G-o-F protein expressions. We further found that the expression of a newly synthesized MOPV Z protein was not visibly impaired by SAMD9L WT and G-o-F (Fig 7B).

Overall, these results suggest viral-specific behaviors to SAMD9L. While lentiviruses are sensitive to WT and G-o-F SAMD9L restriction, the retrovirus MLV and the arenavirus MOPV are resistant to WT SAMD9L in these conditions, but sensitive to the G-o-F SAMD9L. This suggests a specific sensing or activation of SAMD9L by lentiviruses. It also seems that MOPV was generally less restricted, which may result from the experimental setup and from possible viral evasion mechanism to the SAMD9L-F886Lfs*11 translation shutdown.

## Discussion

In this study, we determined that SAMD9L, but not its paralogue SAMD9, acts as an inhibitor of primate lentiviruses: HIV-1, HIV-2, and several SIVs. Instead, SAMD9L does not target MLV, a virus belonging to the gammaretrovirus genus within the family of *Retroviridae*, nor does it target model viruses of 2 RNA virus families, *Arenaviridae* and *Rhabdoviridae*. Mechanistically, we found that SAMD9L restricts lentiviruses at the translation step and also potentially during viral protein trafficking.

We further showed that the constitutively active G-o-F SAMD9L dramatically impacted HIV-1, MLV, and, to a much lesser extent, MOPV. This suggests that SAMD9L virus-specific effect may involve a differential SAMD9L activation—dependent on its capacity to sense a given viral infection—and on the virus ability to escape from SAMD9L restriction.

Finally, we identified SAMD9L determinants of lentiviral inhibition and of cellular translational repression: (i) an essential SLFN-like box effector domain with key E198/D243 residues; and (ii) a C-terminal autoregulatory domain, previously identified in the context of SAAD/ATXPC patients [2]. Overall, our study enlightens that these genes implicated in life-threatening genetic diseases are involved in antiviral immune defense beyond DNA poxviruses [11,12].

We describe the identification of human SAMD9L as an effector of the anti-HIV activity of the type I IFN response, in the late phases of viral replication. SAMD9L may have been missed from most previous HIV-host factor screens, because the protein is poorly expressed in immortalized cell lines used in these screens; except THP-1 myeloid cells in which OhAinle and colleagues had SAMD9L as a potential candidate [19]. Furthermore, SAMD9L antiviral effect is mild against several HIV-1 lab strains, which are tested in most screens, and we revealed its strong antiviral effect using HIV-1 IMCs from primary HIV-1 T/F strains. Understanding the molecular details for SAMD9L regulation, the viral determinants involved in the virus specificity, and the basis for the dichotomy between SAMD9L/HIV-1 inhibition and SAMD9/HIV-1 enhancement will be the next outstanding questions. Because 2 HIV-1 T/F primary strains showed strong sensitivity to SAMD9L restriction, it would be interesting to test more HIV-1 T/F strains, as well as HIV-1 strains from different stages of infection, to determine whether HIV-1 evolves and overcomes SAMD9L restriction over time within people living with HIV and by which mechanism. Finally, defining endogenous SAMD9L as a bona fide restriction factor against HIV would require full validation in primary HIV target cells in knock-out conditions. Overall, our study on the different steps of HIV-1 replication identified that SAMD9L mostly affects lentiviral replication by inhibiting viral protein expression, likely through the reported translational shutdown without affecting HIV-1 genomic and messenger RNA quantity and splice forms. It also potentially affects Env incorporation in virions by its effect on endosomal trafficking and receptor recycling [4,5]. Lastly, although SAMD9 has an opposite proviral effect on HIV-1, it seems to modulate the same HIV-1 replication steps.

We identified 3 acidic residues E186/E198/D243 in the AlbA2 domain of SAMD9L that are similar to the SLFN-box and could constitute a ribonuclease active site. We further demonstrated that this site has the necessary determinants for SAMD9L antiviral and cellular effector functions. The crystal structure of SAMD9 AlbA2 solved in complex with DNA [14] showed the capacity of SAMD9/9L to bind dsNA. One primary hypothesis is, therefore, that SAMD9L AlbA2 effector domain may bind and cleave tRNAs through the E186/E198/D243 active site, similarly as SLFN11, SLFN12, or SLFN13 [27,29,31,33]. Interestingly, SAMD9L and SLFN11 are also both involved in DNA damage response, a function mediated by type II tRNAs cleavage in SLFN11 [1,33]. Other hypotheses are that SAMD9L effect may be linked to mRNA stability or ribosomal protein disruption [34], or given the electrostatic environment of E198/D243, it is possible that the residues convey a proper orientation for R221, allowing its interaction with the dsNA phosphate backbone for efficient binding [14,27]. This latter would be more similar to SLFN5, which only binds tRNA without cleavage [35]. A study, on the poxvirus-restriction activity of SAMD9 published in June 2023, independently identified—and through a different approach—the same active site and showed that SAMD9 is a ribonuclease cleaving phenylalanine tRNAs, which would be associated to ribotoxic stress [36]. In our settings, SAMD9L ribonuclease activity did not lead to apparent HIV-1 RNA degradation. Furthermore, this conserved effector function would not by itself explain the viral specificity. But whatever SAMD9 and SAMD9L exact functions, the identification of these host determinants is of additional importance, as we show that targeting them allows to revert the effect of a pathogenic G-o-F SAMD9L variant from patients with SAAD or associated myeloid disorders, which would therefore have direct clinical relevance.

Importantly, SAMD9 and SAMD9L are both antiviral against poxviruses, whereas only SAMD9L is antiviral against HIV. While sharing only 60% amino acid similarity, the paralogs harbor high structural homology. A hypothesis to explain their divergent effect regarding HIV may reside in the lentiviral escape of SAMD9 antiviral activity through an HIV antagonist or through viral evasion. Because we observe a slight proviral effect of SAMD9 on HIV-1, it would be interesting to investigate whether HIV-1 has even adapted to usurp the IFN-inducible SAMD9 protein as a host proviral factor. This may be similar to influenza virus, which is able to repurpose the IFN-induced IFIT2 to promote viral protein translation [37]. Alternatively, there may be intrinsic functional differences between SAMD9 and SAMD9L, for example, it is possible that SAMD9L is able to sense and/or restrict HIV, as opposed to its paralog. In these cases, during the SAMD9/9L divergence, paralogous genes may have (i) retained similar functions against poxviruses, allowing increased antiviral potency—such as observed for the antiviral IFITM2 and IFITM3 [38,39], or for the antiretroviral primate APOBEC3s [40,41]; and (ii) specialized, with SAMD9L bearing antilentiviral functions, allowing antiviral breadth and diversification of the effector functions—as observed for the mammalian MX, IFIT, or SLFN gene families [15,26,39,42,43]. Similarly to these antiviral host restriction factors, SAMD9 and SAMD9L have both evolved under positive selection in mammals [44], reflecting potential adaptation to past viral epidemics and their importance in viral restriction over evolutionary times [45–47]. These past “evolutionary arms-races” may have involved conflict with ancient poxviruses [11], as well as lentiviruses and possibly other viral families, leading to functional divergence of the paralogs.

Further structural homology analyses on full-length SAMD9L allowed us to identify, in the intermediate/C-terminal region, a homology with the protein PH0952 from archaea *Pyrococcus horikoshii* harboring a double-inhibition mechanism with (i) a signal transduction ATPase with numerous domains (STAND), analogous to apoptotic peptidase activating factor 1 (APAF-1), as previously reported [3,24], but (ii) also a tetratricopeptide repeat (TPR) sensor domain [39]. Thus, the intermediate/C-terminal region of SAMD9L would include at least 2 regulatory mechanisms, possibly self-guarding an accidental activation or exposure of the SLFN-like box (Fig 6A). These types of regulatory mechanisms may have ancient evolutionary roots shared between eucaryotes, archaea, and bacteria [48]. In accordance with previous models [3], the F886LFs11* truncation would lead to a constitutive cellular and antiviral translational shutdown. Therefore, while the N-terminal region contains the necessary and sufficient effector domain for SAMD9L-driven translational repression, the C-terminal region could provide strong self-guarding, whose mechanism and evolutionary origin remain to be characterized. In this work, we started to identify some virus specificity towards SAMD9L, where the lentivirus HIV-1 is restricted by human SAMD9L, while the gammaretrovirus MLV and the arenavirus MOPV do not activate SAMD9L but can be restricted by the constitutively active SAMD9L. Because MOPV is only mildly affected by the G-o-F SAMD9L, it is possible it further antagonizes SAMD9L or, more probably, MOPV may use specific mechanisms of translation that are less sensitive to SAMD9L activity, allowing some evasion from restriction.

Although distantly related, IFN-stimulated SAMD9/9L and SLFN proteins bear some redundant and crucial cellular and cell-autonomous innate immune antiviral functions. Both proteins are differentially, but tightly, autoregulated like many genes involved in type I interferonopathies [18]. Malfunction in these gene families can lead to dramatic human genetic and infectious diseases and underlie a determinant role for their proper regulation in the cell, which require further mechanistic and translational research.

### Limitation of the study

This study could only address the physiological relevance of the SAMD9L effect on the virus side, using natural HIV-1 strains. On the host side, we could not obtain CRISPR-KO of endogenous SAMD9L in primary CD4+ T cells, possibly due to toxicity. Therefore, despite multiple lines of evidence that SAMD9/9L are important antiviral defense factors, the extent of their antiviral breadth and the full characterization of SAMD9L effect in primary HIV-1 target cells and from naturally infected individuals are still required to identify SAMD9L as a bona fide antiviral HIV restriction factor and a broad antiviral cell autonomous immune defense.

## Materials and methods

### Cell lines and culture

Human embryonic kidney 293T (ATCC, cat. CRL-3216), HeLaP4P5 (HeLa cells expressing CD4^hi^CCR5^hi^CXCR4^hi^), and TZM-bl (NIH AIDS Research and Reference Reagent Program, Cat. 8129) cells were maintained in Dulbecco’s Modified Eagle Medium (DMEM) supplemented with 10% fetal calf serum (FCS; Sigma cat. F7524) and 100 U/ml of penicillin/streptomycin. TZM-bl cells express CD4, CCR5, and CXCR4 and encode for luciferase and β-galactosidase under the LTR promoter. They are routinely used for titration of lentiviral supernatants. SupT1 T lymphocytic cell lines were maintained in RPMI-1640 with 10% FCS. Primary blood monocytes and lymphocytes were purified from leukopacks of healthy blood donors, using Ficoll and Percoll gradients. Lymphocytes were activated for >24 h with 1 μg/ml PHA (Sigma) and 150 U/ml IL2 (Eurobio, PCUT-209). Monocytes were purified using Miltenyi monocyte isolation kit II (cat. 130-091-153) and differentiated in macrophages upon incubation for >4 days in complete RPMI-1640 with 10% FCS and 100 ng/ml Macrophage-Colony Stimulating Factor (M-CSF, Eurobio, cat. 01-A0220-0050).

### Plasmids

The virus and host plasmids used in this study are shown in S1 Table. SAMD9L-E198A/D243A, SAMD9L-F886Lfs*11, and SAMD9L-F886Lfs*11-E198A/D243A were generated from pMAX:mTFP1-SAMD9L plasmid using the QuikChange Lightning Site-Directed Mutagenesis Kit (Agilent) following the manufacturer’s instructions. Similarly, the Chinese hamster chSAMD9L-D237A was generated from the original chSAMD9L plasmid (pcDNA3.1/V5-His-Topo:3xFLAG-chSAMD9L). The frameshift F886Lfs*11 was generated by deleting 2 threonines.

### Replication-competent lentivirus production and titration of the infectious virus yields

293T cells were seeded in 6-well plates at 0.2M cells/ml. Twenty-four hours later, cells were cotransfected with TransIT-LT1 (Mirus) with a plasmid encoding a fully replication-competent lentivirus (IMCs), as well as a plasmid encoding either SAMD9, SAMD9L, or an empty control. In the case of antiretroviral protease inhibitor treatment, cells were treated 15 min prior to transfection with 10 mM Saquinavir and 1 mM Indinavir. Unless stated in the text or figure (e.g., for viral and host doses), the DNA quantity was 1,500 ng for the host plasmids and 1,200 or 1,600 ng for the virus plasmids. Forty-eight hours posttransfection, the cells were harvested for western blot or qPCR analyses. The supernatants were collected and stored at −80°C for further analyses, including titration of infectious virus yield by TZM-bl cells, titration of viral proteins by ELISA p24/p27, or western blot analysis of virions after ultracentrifugation. For infectious virus yield titration, TZM-bl cells were seeded in 96-well plates and infected by a serial dilution of viral supernatant. Forty-eight hours postinfection, cells were lysed using BrightGlow Lysis Reagent (Promega E2620), and the relative luminescence units (RLUs) were measured by Tecan Spark Luminometer. Infectious virus yields of viruses in various conditions are always expressed as fold-change as compared to a paired viral infection condition in the absence of any SAMD9/9L.

### Virus release titrations by ELISA

Titration of HIV-1, as well as HIV-2 and SIV, viruses in the supernatant were performed using HIV-1 p24 and SIV p27 ELISA kits (XpressBio), respectively, following the manufacturer’s instructions.

### Western blotting

Cells were harvested, lysed by ice-cold RIPA buffer (50 mM Tris (pH 8), 150 mM NaCl, 2 mM EDTA, 0.5% NP40) supplemented with protease inhibitors (Roche) and by sonication. For the supernatant fraction, 1 ml of HIV-1 replication-competent supernatant was collected and purified through 200 μl sucrose (25%) by ultracentrifugation, and the pellets were resuspended in ice-cold RIPA buffer. Proteins from cell lysates or from supernatant were then similarly processed: separated by electrophoresis and then transfected on PVDF membrane through a wet transfer overnight at 4°C. In most cases, a Stain-Free gel (BioRad), which allows to mark total protein expression, was used and allowed for loading and protein transfer controls. Importantly, because SAMD9/9L are known to affect protein synthesis, this was the most robust way to control for protein loading and total protein content, instead of only checking for 1 housekeeping gene (such as actin or tubulin). The membranes were blocked in a TBS-T 1X solution (« Tris Buffer Saline », Tris HCl 50 mM (pH 8), NaCl 30 mM, 0.05% of Tween 20) with 5% powder milk and were incubated for 1 h to overnight in primary antibodies and 1 h in secondary antibodies. Detection was made with SuperSignal West Pico Chemiluminescent Substrate (Thermo Fisher Scientific) or Clarity ECL substrate (BioRad) using the Chemidoc Imaging System (BioRad). For endogenous expression of SAMD9 and SAMD9L, cells were stimulated, or not, with IFN⍺2 (Tebu Bio, cat. 11100–1) or IFNβ (500 IU/ml, R&D Systems, 8499-IF-010) for 24 h prior lysis.

The following antibodies were used: anti-Tubulin (Sigma, cat. T5168), anti-Actin (Sigma, cat. A2228), anti-Flag (Sigma, cat. F3165), anti-SAMD9L (Proteintech, 25173-1-AP), anti-SAMD9 (Sigma, HPA021319), anti-HIV-1-Gag (NIH HIV Reagent Program, 183-H12-5C), anti-HIV-1-gp120 (Aalto, D7324; NIH HIV Reagent Program, 16H3), rabbit primary antibodies specific to the MOPV Z protein (Agrobio), secondary anti-mouse and anti-rabbit IgG-Peroxidase conjugated (Sigma, cat. A9044 and AP188P, respectively). “Total protein” was also used as loading control, using Stain-Free gel (BioRad). The image of uncropped gels and membranes, corresponding from the cropped version in the Figures, are presented in S1 Raw Images.

### Viral-like particle (VLP) production and infection for single-round GFP- or luciferase-reporter HIV-1, SIVmac, and MLV

For production, 293T cells were transfected by calcium phosphate method with DNA plasmids encoding the packaging plasmid, the genome plasmid, and the envelope plasmid (VSVg, pMD2.G) at ratios of 2:2:1. For HIV-1 VLPs, the packaging plasmid was pHIV-1 GagPol 8.2 (that includes most viral accessory genes) or psPAX2 (devoid of accessory genes), and the reporter genome plasmid was pHIV-1-LTR-fLuc. For SIVmac VLPs, the packaging plasmid was pSIV3+ (that includes most accessory genes), and the reporter genome plasmid was pGAE-fLuc. For MLV VLPs, the packaging pMLV plasmid was pTG5349 and the reporter plasmid pMLV-CMV-GFP was pTG13077. Details and references of plasmids are available in S1 Table. Two to three days posttransfection, the viral supernatant was filtered through a 0.45-μm diameter filter or purified by ultracentrifugation. Supernatant was then used for titration by exogenous RT activity (10 μl), p24/p27 ELISA (10 μl), and used for subsequent infection of 293T cells in serial dilutions.

To assess the effect of SAMD9L on the early phases of lentiviral replication, different amounts of purified Luc-VLPs were used to infect 293T cells, which were transfected 24 h or 48 h before with either empty, SAMD9, or SAMD9L plasmid. Two days postinfection with Luc-VLPs, cells were lysed with BrightGlow Lysis Reagent (Promega E2620) and the mean RLUs were directly measured by FLUOStar Optima reader.

To assess the effect of SAMD9L on the late phases of MLV replication, the MLV-VLP:GFP plasmids were cotransfected along with empty or SAMD9L plasmids, and supernatant was collected 2 to 3 days posttransfection for titration. Infectious virus yield was quantified by infecting 293T cells. Cells were trypsinized 2 days postinfection and fixed in PFA before quantification on BD FACSCanto II (SFR BioSciences) and analyses in FlowJo.

### Cell counting

293T cells were seeded in 6-well plates at 0.2M cells/ml and were transfected with TransIT-LT1 (Mirus) 24 h later with 1,500 ng of empty plasmid, or of plasmid encoding SAMD9 or SAMD9L, as in other experiments. Cells were counted with trypan blue at 0, 24, 48, and 72 h posttransfection, corresponding to the maximum period during which the cells are maintained for virus replication experiments. The experiment was performed in triplicates in 3 independent biological replicates. For the analyses, the cell counts of SAMD9 and SAMD9L conditions were normalized to the control condition.

### HIV-1 replication in SAMD9L knock-down cells

The shRNA oligos were designed from the Broad Institute Genetic Perturbation platform, and the shRNA clones in pLKO.1 were purchased from Sigma. The 3 oligo sequences for pLKO.1-shSAMD9L are: 5′- CCGGGCAGACAGTATTGCACTAAATCTCGAGATTTAGTGCAATACTGTCTGCTTTTTTG, 5′- CCGGCATCGCTACATAGAACATTATCTCGAGATAATGTTCTATGTAGCGATGTTTTTTG, 5′- CCGGGCTCTTATGTTACTGACTCTACTCGAGTAGAGTCAGTAACATAAGAGCTTTTTTG. shRNA oligos targeting CD241 (also named RHAG) were controls as in [23]: 5′- CCGGGCAAGAATAGATGTGAGAAATCTCGAGATTTCTCACATCTATTCTTGCTTTTTG, 5′- CCGGCCTCTGACATTGGAGCATCAACTCGAGTTGATGCTCCAATGTCAGAGGTTTTTG, 5′- CCGGGATGACAGGTTTAATTCTAAACTCGAGTTTAGAATTAAACCTGTCATCTTTTTTG. shRNA-coding lentivectors were prepared in 10-cm dishes by transfection of 293T cells, using calcium phosphate method, of 2.5 μg of psPAX2, 0.75 μg of VSVg, and 1 μg of each shRNA-coding construct. After 72 h, the supernatant was collected and purified by ultracentrifugation, and the shRNA lentiviral stock was titrated by HIV-1 p24 ELISA (XpressBio). To generate HelaP4P5 SAMD9L KD and control (CD241) KD, the cells were plated at 0.2M cells/ml in a 6-well plate and stimulated with IFNβ (500 IU/ml, R&D Systems cat. 8499-IF-010). At 24 h, cells were transduced with 100 ng p24 of shRNA lentiviral particles targeting SAMD9L or CD241. After 48 h, cells were passaged under puromycin selection. The expression of SAMD9L in shSAMD9L and shControl cells was controlled by western blot before HIV-1 replication. For HIV-1 replication, shSAMD9L and shControl cells were seeded in 12-well plates, with IFNβ treatment, and were infected in technical duplicates the next day with the equivalent of 10 ng Gag p24 (HIV-1 p24 ELISA) of replication-competent HIV-1 LAI, HIV-1 pCH077, or HIV-1 pCH077 pseudotyped with VSVg for the first round of infection. Media were changed 6 h postinfection. Supernatant was then collected at day 0, 2, 4, and 6 postinfection and titrated by infection in TZM-bl assay. Each condition was performed in duplicates in a total of 2 to 3 independent biological replicates.

### Protein synthesis assay by flow cytometry

293T cells were seeded at 0.3M cells/ml in 12-well poly-L-lysine coated plates. Twenty-four hours later, medium was changed, and cells were transfected with TransIT-LT1 (Mirus) with 3 μg of vector encoding SAMD9L or pcDNA3.1 empty. Seventy-two hours posttransfection, cells were incubated in OPP (Immagina Biotechnology) for 30 min at 37°C. Medium was discarded, and cells were trypsinized, harvested, and fixed with PFA 4%. Cells were then washed with PBS BSA 3% and permeabilized in PBS 0.5% Triton X-100 for 15 min. Click-iT Plus Alexa Fluor Picolyl Azide assay was then performed, and cells were analyzed on MACSQuant VYB Cytometer (Miltenyi Biotec, SFR BioSciences).

### RT-qPCR

293T cells prepared for replication-competent lentiviral production, and infection were harvested and lysed by TRIzol Reagent (Thermo Fisher Scientific). Total RNA was extracted and purified following the manufacturer’s protocol. RNA was treated with RQ1 RNase-Free DNase (Promega) following the manufacturer’s protocol. The RT step was performed using the SuperScript III First-Strand Synthesis System (Invitrogen). FastStart Universal SYBR Green Master (Roche) was used for qPCR and was performed on StepOnePlus Real-Time PCR System (Thermo Fisher Scientific, SFR BioSciences). Primer sequences were designed to target Gag and LTR from lentiviruses and TBP and U6 from the host (as controls); sequences are available in S2 Table. For Gag and LTR RNA quantification, double normalization was performed using TBP and U6. For TBP quantification, U6 normalization was used and vice versa.

### VSV infections

293T cells were seeded in 12-well plate and were transfected 24 h later with 800 ng of SAMD9L-encoding plasmid, ISG20-encoding plasmid [22] as a positive control for VSV restriction, or an empty plasmid as a negative control. Twenty-four hours posttransfection, cells were infected with VSV-GFP [49] at MOI 0.3, and, 16 hours postinfection, the viral supernatant was collected for each condition and titered on new cells through the GFP reporter. Single-cell analysis was acquired with BD FACSCanto II Flow Cytometer (SFR BioSciences). The percentage of VSV-GFP+ cells in the absence of SAMD9L or other host factor (empty control) was used as the control and the result of the other conditions are expressed as fold changes normalized to the empty control. Each condition was performed in technical duplicates, and the experiments were performed in 4 independent biological replicates.

### Mopeia virus (MOPV) infections

293T cells were seeded in 24-well plates at 0.2M cells per well. The following day, 1 μg of the SAMD9L plasmid was transfected with Lipofectamine 2000 (Thermo Fisher Scientific), following the manufacturer’s instructions. In addition, 1 μg of a plasmid coding for mCherry was used as a transfection positive control and verified by fluorescence microscopy before 293T infection. For infection, 293T cells expressing mCherry or SAMD9L were infected with a recombinant MOPV (MOPV_wt_) [50] at an MOI of 0.01. Inocula were prepared by diluting viruses in DMEM 2% FCS, 0.5% P/S. Cell supernatants were removed and viral inocula were added to corresponding wells. After a 1-h incubation, wells were washed and reincubated for 48 h. The cell supernatants of each condition were then collected and frozen at −80°C. Cells were collected and lysed in 2X Laemmli buffer and analyzed by western blot to control for the expression of SAMD9L. For the titration of MOPV, Vero E6 cells were seeded in 12-well plates at 0.25M cells per well in DMEM 5% FCS, 0.5% P/S. The following day, cells were incubated with successive decimal dilutions of each sample supernatant for 1 h at 37°C and 5% CO_2_. After an hour, 1.5 mL of a carboxymethylcellulose (CMC) solution was added to each well, and plates were incubated at 37°C, 5% CO_2_ for 7 days. CMC was then removed and cells were fixed in PBS 4% formaldehyde and permeabilized in PBS 0.5% Triton X-100. Focal forming units (FFUs) were detected using rabbit primary antibodies specific to the MOPV Z protein (Agrobio) and secondary alkaline phosphatase-conjugated antibodies (Sigma-Aldrich Merck). Plates were revealed using an NBT/BCiP solution (Thermo Fisher Scientific), and foci were counted to determine the virus titers. Viral titers were expressed as FFUs per milliliter (FFU/mL).

### Structure homology analyses

HHpred was used for protein homology detection analyses [51]. Protein structures were retrieved from AlphaFold DB and RCSB PDB [52–54]. Structural analyses and visualization were performed using UCSF Chimera (The Regents) and PyMOL Molecular Graphic Systems (Schrödinger, LLC) softwares.

### Host and viral sequence analyses

SAMD9 and SAMD9L orthologs from mammals were retrieved from public online databases using NCBI BLASTn with the human sequences of SAMD9 and SAMD9L as queries. SLFN sequences were retrieved using NCBI. HIV and lentivirus sequences were retrieved using the HIV sequence database http://www.hiv.lanl.gov/. Amino acid alignments were performed using Muscle [55]. Geneious (Biomatters) and ESPript 3.0 [56] were used for sequence displays.

### Other softwares and statistical analyses

Sequencing analyses and representations were performed in Geneious (Biomatters). Quantification of images (western blot analyses) were performed using Image Lab (BioRad). Graphic representations and statistics were performed using GraphPad Prism 9 (except some with Excel from Microsoft Office). In Figures, data are represented as mean ± SD. Ratio paired *t* test was performed for Figs 1B, 1D–1F, 1H, 2, 3, 4, 6, and 7A. Paired t test was performed for Figs 1G, 5, and 7B.

## Supporting information

S1 Fig SAMD9/9L ectopic expression in lentiviral producer cells and impact on total protein expression. (**A**) Western blot analysis of SAMD9/9L and tubulin expression from lysates of the lentiviral producer cells in the context of ectopic SAMD9/9L expression (1,500 ng input DNA). (**B**) Mean of 4 independent biological replicates for the quantification of total protein expression in cells ectopically expressing SAMD9 or SAMD9L in the experimental setting presented in Fig 2A, normalized to the control. Quantifications are based on Stain-Free gel (BioRad) images using Image Lab software. The data and the raw images underlying this Figure can be found in S1 Data and S1 Raw Images.
(PDF)

S2 Fig SAMD9L is a specific antiviral factor of HIV-1. (**A**, **B**) HIV-1 infectious yields from +/− SAMD9L expressing cells according to the viral doses. Infectious yield of HIV-1 pCH077, pWITO, and LAI from supernatant of 293T cells cotransfected with empty plasmid (control) or 1,500 ng of SAMD9L plasmid and with 400, 800, 1,200, or 1,600 ng of IMCs. Infectious virus yield was measured by luminescence (RLU) in the TZM-bl cell assay (Fig 1A). Raw RLU for the 3 independent biological replicates (Rep. 1–3; panel A) and corresponding relative infectious virus yields (mean of panel A with a normalization to the control (in black) (panel B). (**C**) HIV-1 infectious yield from +/− SAMD9/9L expressing cells according to the SAMD9/9L doses. Relative infectious virus yield of HIV-1 pCH077 and LAI from cells expressing SAMD9L or SAMD9 normalized to the control. 293T cells were cotransfected with empty plasmid (control) or 400, 1,000, or 1,500 ng of SAMD9L or SAMD9 plasmid, and with HIV-1 pCH077 or LAI plasmid. (**D**) 293T cells were cotransfected with 1,500 ng of SAMD9L or empty plasmid (control), and with plasmids to encode for MLV:VSVg pseudoviruses: pTG5349 (MLV gagpol), PTG13077 (MLV LTR-GFP), and pMD2.G (VSVg). Viral supernatant was collected 48 h later, and 0, 5, 10, 50, and 100 μl of supernatant was used to infect new target cells. Two days later, infectious virus yield was measured by FACS (% of GFP-positive cells) in the control (in black) and SAMD9L (in blue) conditions. Results are shown for 3 independent biological replicates. Fig 1F is the mean of these replicates. (**E**) Same as D with a dose of MLV (400, 800, 1,200, or 1,500 ng of DNA MLV gagpol plasmid). Cells were infected with 25 μl of supernatant. No difference between SAMD9L and control conditions (*p* > 0.05). The data underlying this Figure can be found in S1 Data. HIV-1, human immunodeficiency virus type 1; IMC, infectious molecular clone; MLV, murine leukemia virus; RLU, relative luminescence unit; SAMD9, sterile alpha motif domain-containing protein 9; SAMD9L, sterile alpha motif domain-containing protein 9-like.
(PDF)

S3 Fig HIV-1 and SIV Gag and Env protein expression in the supernatant and in the producer cells are restricted by SAMD9L. (**A**) Titration by ELISA p24 Gag of HIV-1 LAI in the supernatant of the producer cells overexpressing or not SAMD9L in the context of a dose of pLAI IMC plasmid (400, 800, 1,200 ng). (**B**) Western blot analyses of HIV-1/SIV Gag and Env from lysates of the lentiviral producer cells in the context of overexpressed SAMD9/9L (1,500 ng). This western blot was also used in S1A Fig for SAMD9/9L. Quantification of p24 normalized to tubulin are presented below the corresponding lanes. SIVmac Env is not detectable with HIV-1 Env antibody. The data and the raw images underlying this Figure can be found in S1 Data and S1 Raw Images. HIV-1, human immunodeficiency virus type 1; IMC, infectious molecular clone; SAMD9/9L, sterile alpha motif domain-containing proteins 9 and 9-like; SIV, simian immunodeficiency virus.
(PDF)

S4 Fig Effect of SAMD9L on HIV-1 protein expression upon lentiviral doses: HIV-1 Gag and Env protein productions are restricted by SAMD9L. Western blot analyses of HIV-1 Env and Gag proteins in the viral producer cells (**A**) and in the supernatant (**B**). Cells were cotranfected with empty plasmid (control) or 1,500 ng of SAMD9L plasmid and with 400, 800, 1,200, or 1,600 ng of IMC for pLAI and pWITO (only 800 and 1,200 ng pWITO conditions are shown in the cell lysates). Quantifications are presented below the corresponding lanes for Env and Gag p24 protein expression, normalized to the total proteins in the cell fraction, and expressed as fold difference compared to the condition in the absence of SAMD9/9L normalized to 1. The raw images underlying this Figure can be found in S1 Raw Images. HIV-1, human immunodeficiency virus type 1; IMC, infectious molecular clone; SAMD9/9L, sterile alpha motif domain-containing proteins 9 and 9-like.
(PDF)

S5 Fig SAMD9L does not affect the early phases of primate lentivirus replication. (**A**) Experimental setup (d1, day 1). Briefly, single-round lentiviral particles, HIV-1:VSVg-Luc and SIVmac:VSVg-Luc, were produced in 293T cells. Two days later, viral supernatant was collected and 1, 2, 10, or 50 μl was used to infect target cells overexpressing or not SAMD9L. Two days postinfection, cells were lysed and a luciferase assay was performed for titration. (**B**) Infectious virus yield to assess the impact of SAMD9L on the early phases of lentiviral replication. The Y-axis shows the RLUs in the luciferase assay. The data represent the mean of 3 independent experiments. (**C**) Corresponding relative infectious virus yield of HIV-1:Luc and SIVmac:Luc (from the 10 μl condition in B), normalized to the control condition (100%). RLU, relative luminescence unit; SAMD9L, sterile alpha motif domain-containing protein 9-like.
(PDF)

S6 Fig Endogenous interferon-stimulated SAMD9L restricts HIV-1 replication. Experimental setup for the KD and viral replication experiments is shown in Fig 3B. Results from the replication experiments of HIV-1 LAI, HIV-1 T/F pCH077, and HIV-1 pCH077:VSVG in shControl or shSAMD9L HeLaP4P5 stimulated with IFNβ over 6 days of viral replication. Titration of the supernatant was performed using the TZM-bl assay at day 2, 4, and 6. Here are presented normalized means of the 3 independent biological replicates for HIV-1 pCH077 and pCH077:VSVg, and 2 for LAI (normalized to the titers in shControl conditions). The data underlying this Figure can be found in S1 Data. HIV-1, human immunodeficiency virus type 1; KD, knock-down; SAMD9L, sterile alpha motif domain-containing protein 9-like; T/F, transmitted/founder.
(PDF)

S7 Fig SAMD9 homology with SLFN proteins. (**A**) SAMD9 crystal structure in complex with DNA (PDB 7KSP). Atoms of the residues forming the active site are represented with purple sticks (labeled in black), while those involved in DNA binding are in yellow. (**B**) Snapshot of the alignment of rSLFN13 and SAMD9L from HHpred analysis results. (**C**) Structural overlap of SLFN N-Domain including the SLFN-box of SLFN5, SLFN12, SLFN13, and SAMD9 AlbA2 domain. Residues forming the active site are indicated in purple below the schematic representation of SLFN family’s domains. (**D**) Representation of the SLFN-box, including the putative active site in purple, of SLFN5, SLFN12, SLFN13, and SAMD9. Coordinates are labeled in black. SAMD9, sterile alpha motif domain-containing protein 9; SAMD9L, sterile alpha motif domain-containing protein 9-like; SLFN, Schlafen.
(PDF)

S1 Table Plasmids used in the study. (DOCX)

S2 Table Primers for qPCR used in the study. (DOCX)

S1 Data Raw data associated with the manuscript. (XLSX)

S2 Data FCS files and gating strategy associated with the manuscript. (ZIP)

S1 Raw Images Raw uncropped western blot images. Uncropped membranes and Stain-Free BioRad gels for Figs 1C, 2C, 2D, 3C, 4A, 5F, 6C, 6D, 7A, 7B, S1A, S3B, S4A, and S4B. Corresponding cropped blots are circled in red.
(PDF)

## Abbreviations

AGS: Aicardi–Goutières syndrome
AIDS: acquired immunodeficiency syndrome
APAF-1: apoptotic peptidase activating factor 1
ATXPC: ataxia-pancytopenia
chSAMD9L: Chinese hamster SAMD9L
CMC: carboxymethylcellulose
DMEM: Dulbecco’s Modified Eagle Medium
dsNA: double-stranded nucleic acid
FCS: fetal calf serum
FFU: focal forming unit
G-o-F: gain-of-function
gRNA: genomic RNA
HIV-1: human immunodeficiency virus type 1
IFN: interferon
IMC: infectious molecular clone
ISG: interferon-stimulated gene
KD: knock-down
MLV: murine leukemia virus
MOPV: mopeia virus
OPP: O-propargyl-puromycin
PBL: primary blood lymphocyte
RLU: relative luminescence unit
RT-qPCR: quantitative reverse transcription PCR
SAAD: SAMD9L-associated autoinflammatory disease
SAMD9/9L: sterile alpha motif domain-containing proteins 9 and 9-like
SIV: simian immunodeficiency virus
SLFN: Schlafen
STAND: signal transduction ATPase with numerous domains
TBP: TATA-binding protein
T/F: transmitted/founder
TPR: tetratricopeptide repeat
VLP: viral-like particle
VSV: vesicular stomatitis virus
WT: wild-type
