# Supplementary figures and images for: SAMD9L acts as an antiviral factor against HIV-1 and primate lentiviruses by restricting viral and cellular translation
Source: PLoS Biol. 2024 Jul 3;22(7):e3002696. doi: 10.1371/journal.pbio.3002696 (PMC11221667; doi:10.1371/journal.pbio.3002696)

A

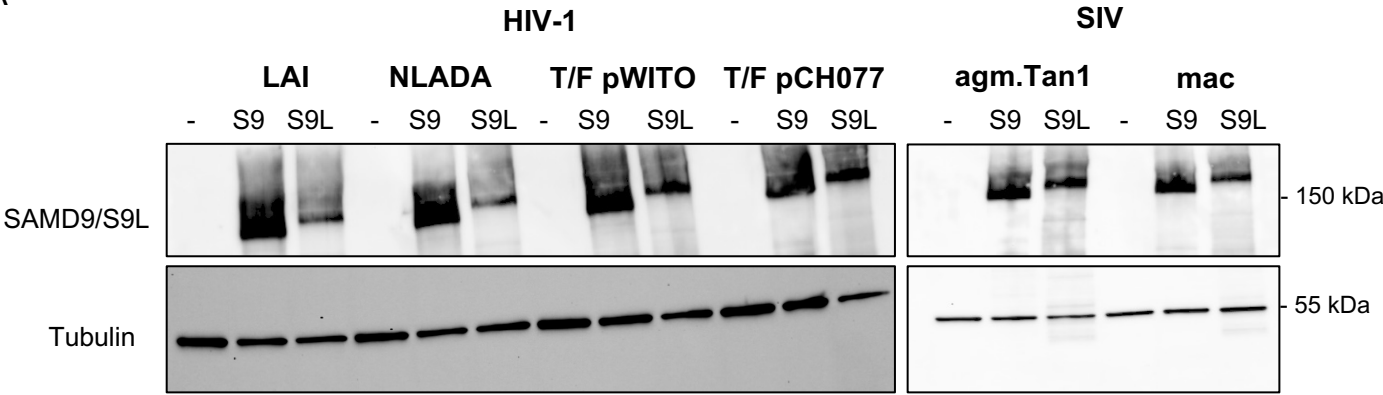

B

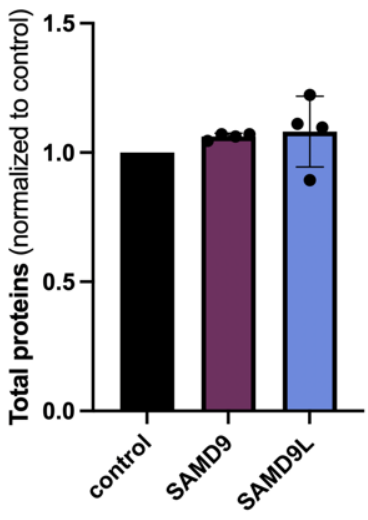

Figure S1

Supplement: S1 Fig — (A) Western blot analysis of SAMD9/9L and tubulin expression from lysates of the lentiviral producer cells in the context of ectopic SAMD9/9L expression (1,500 ng input DNA). (B) Mean of 4 independent biological replicates for the quantification of total protein expression in cells ectopically expressing SAMD9 or SAMD9L in the experimental setting presented in Fig 2A, normalized to the control. Quantifications are based on Stain-Free gel (BioRad) images using Image Lab software. The data and the raw images underlying this Figure can be found in S1 Data and S1 Raw Images. (PDF) [file pbio.3002696.s001.pdf]

# HIV-1 infectious yield according to viral doses

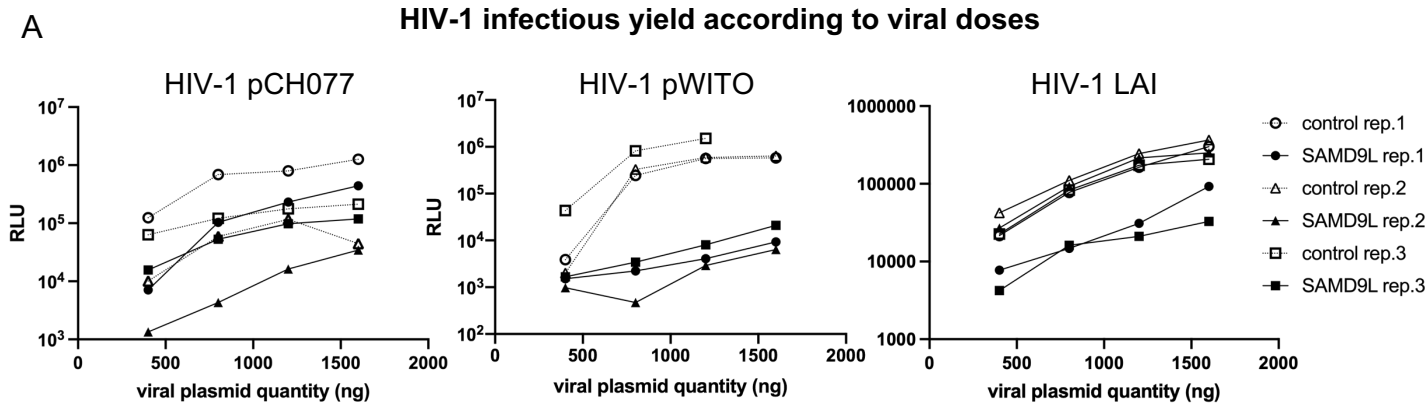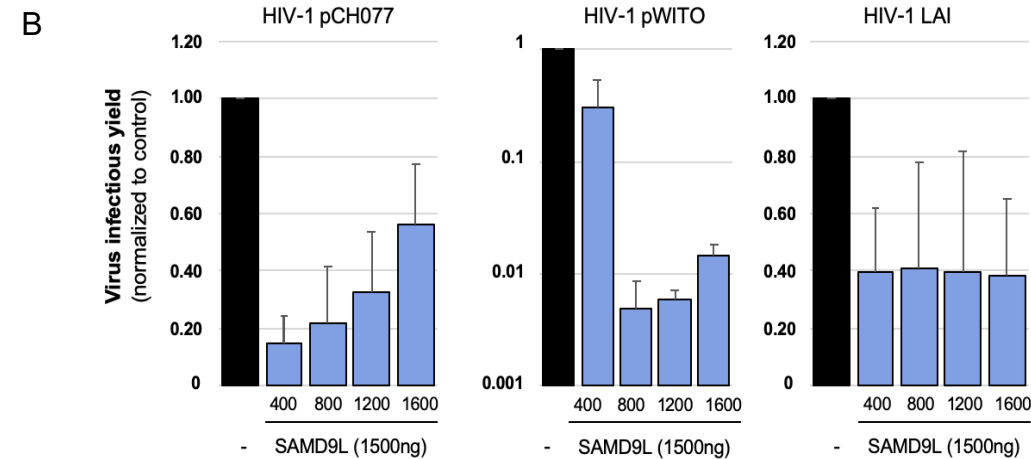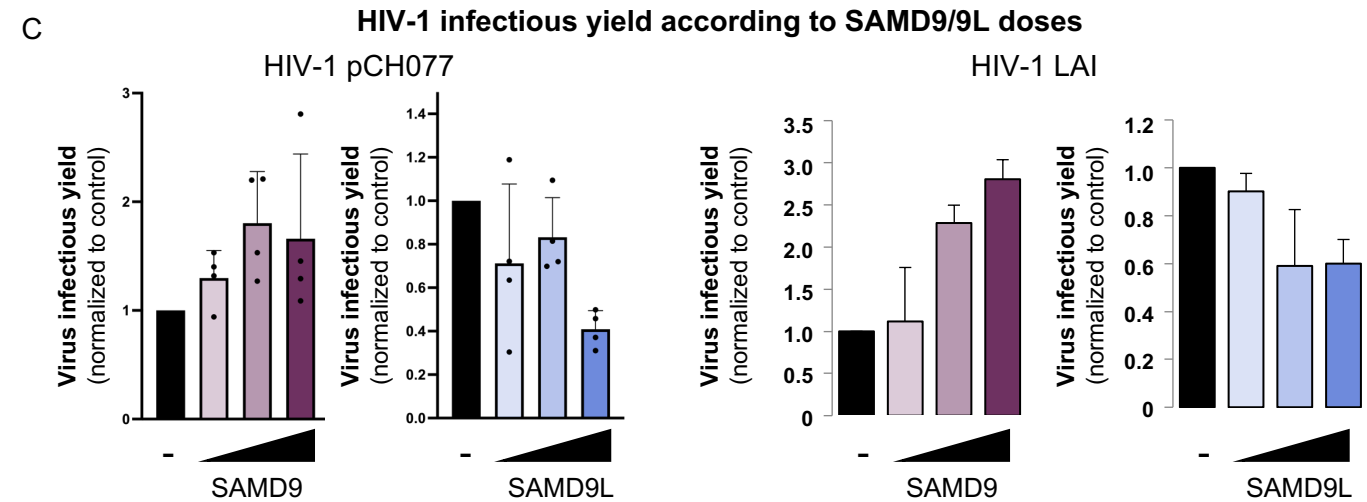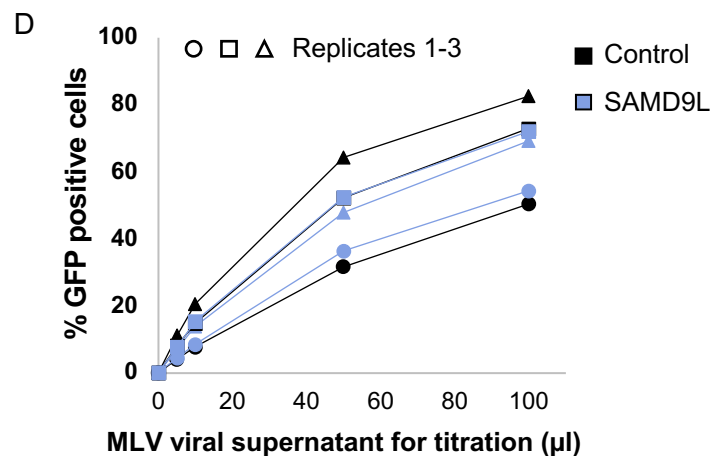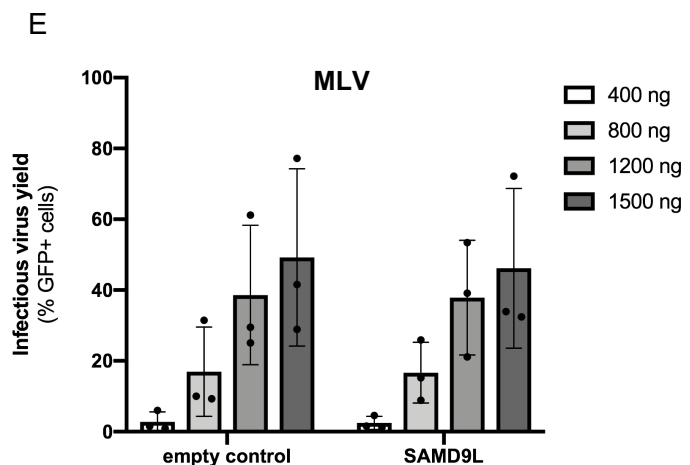

Figure S2

Supplement: S2 Fig — (A, B) HIV-1 infectious yields from +/− SAMD9L expressing cells according to the viral doses. Infectious yield of HIV-1 pCH077, pWITO, and LAI from supernatant of 293T cells cotransfected with empty plasmid (control) or 1,500 ng of SAMD9L plasmid and with 400, 800, 1,200, or 1,600 ng of IMCs. Infectious virus yield was measured by luminescence (RLU) in the TZM-bl cell assay (Fig 1A). Raw RLU for the 3 independent biological replicates (Rep. 1–3; panel A) and corresponding relative infectious virus yields (mean of panel A with a normalization to the control (in black) (panel B). (C) HIV-1 infectious yield from +/− SAMD9/9L expressing cells according to the SAMD9/9L doses. Relative infectious virus yield of HIV-1 pCH077 and LAI from cells expressing SAMD9L or SAMD9 normalized to the control. 293T cells were cotransfected with empty plasmid (control) or 400, 1,000, or 1,500 ng of SAMD9L or SAMD9 plasmid, and with HIV-1 pCH077 or LAI plasmid. (D) 293T cells were cotransfected with 1,500 ng of SAMD9L or empty plasmid (control), and with plasmids to encode for MLV:VSVg pseudoviruses: pTG5349 (MLV gagpol), PTG13077 (MLV LTR-GFP), and pMD2.G (VSVg). Viral supernatant was collected 48 h later, and 0, 5, 10, 50, and 100 μl of supernatant was used to infect new target cells. Two days later, infectious virus yield was measured by FACS (% of GFP-positive cells) in the control (in black) and SAMD9L (in blue) conditions. Results are shown for 3 independent biological replicates. Fig 1F is the mean of these replicates. (E) Same as D with a dose of MLV (400, 800, 1,200, or 1,500 ng of DNA MLV gagpol plasmid). Cells were infected with 25 μl of supernatant. No difference between SAMD9L and control conditions (p > 0.05). The data underlying this Figure can be found in S1 Data. HIV-1, human immunodeficiency virus type 1; IMC, infectious molecular clone; MLV, murine leukemia virus; RLU, relative luminescence unit; SAMD9, sterile alpha motif domain-containing protein 9; SAMD9L, sterile al [file pbio.3002696.s002.pdf]

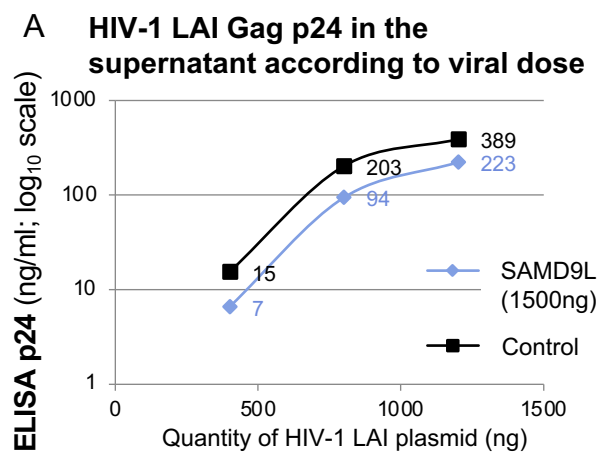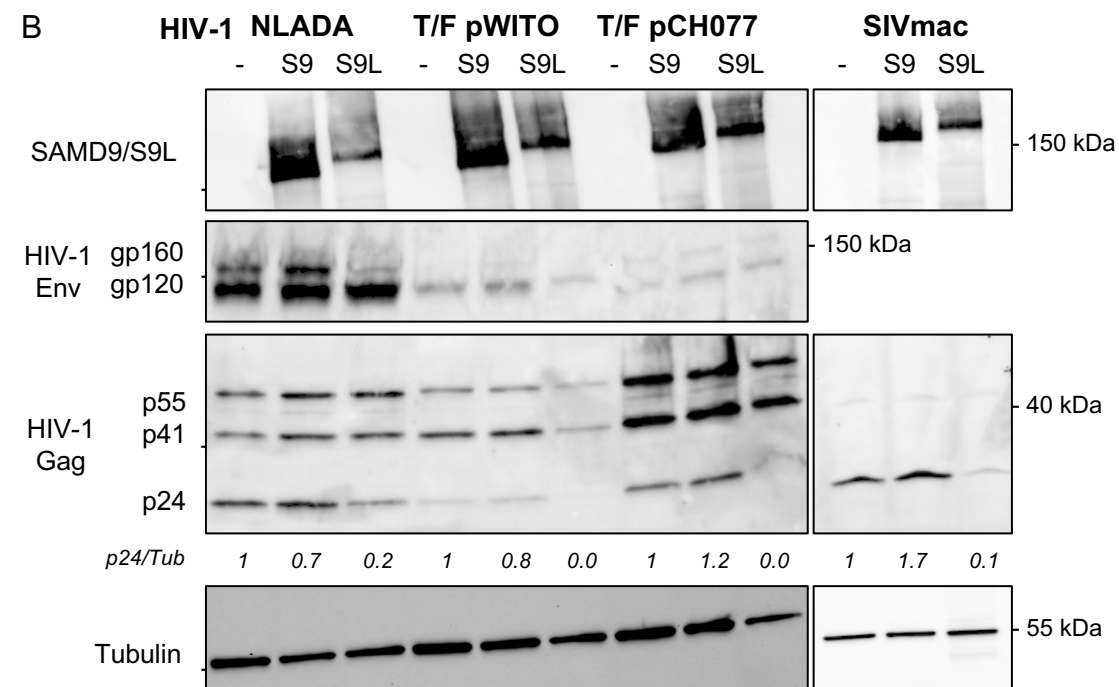

Figure S3

Supplement: S3 Fig — (A) Titration by ELISA p24 Gag of HIV-1 LAI in the supernatant of the producer cells overexpressing or not SAMD9L in the context of a dose of pLAI IMC plasmid (400, 800, 1,200 ng). (B) Western blot analyses of HIV-1/SIV Gag and Env from lysates of the lentiviral producer cells in the context of overexpressed SAMD9/9L (1,500 ng). This western blot was also used in S1A Fig for SAMD9/9L. Quantification of p24 normalized to tubulin are presented below the corresponding lanes. SIVmac Env is not detectable with HIV-1 Env antibody. The data and the raw images underlying this Figure can be found in S1 Data and S1 Raw Images. HIV-1, human immunodeficiency virus type 1; IMC, infectious molecular clone; SAMD9/9L, sterile alpha motif domain-containing proteins 9 and 9-like; SIV, simian immunodeficiency virus. (PDF) [file pbio.3002696.s003.pdf]

A Cell lysates

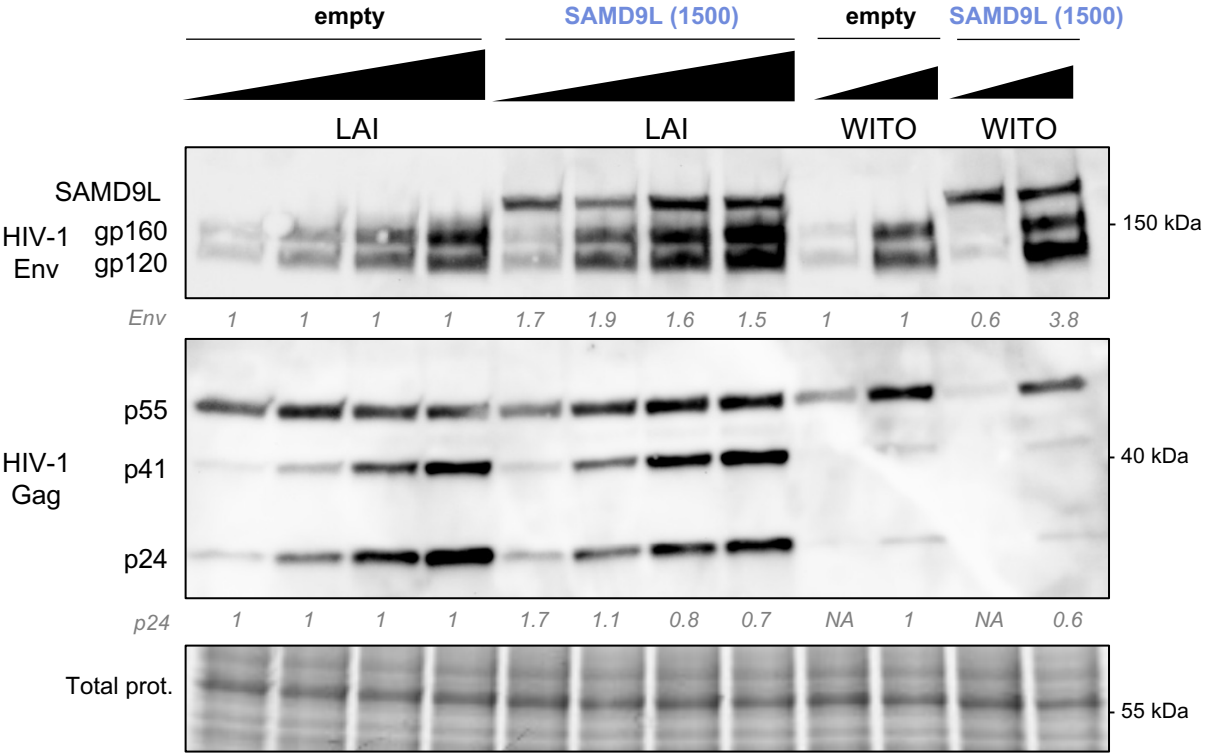

B Virions

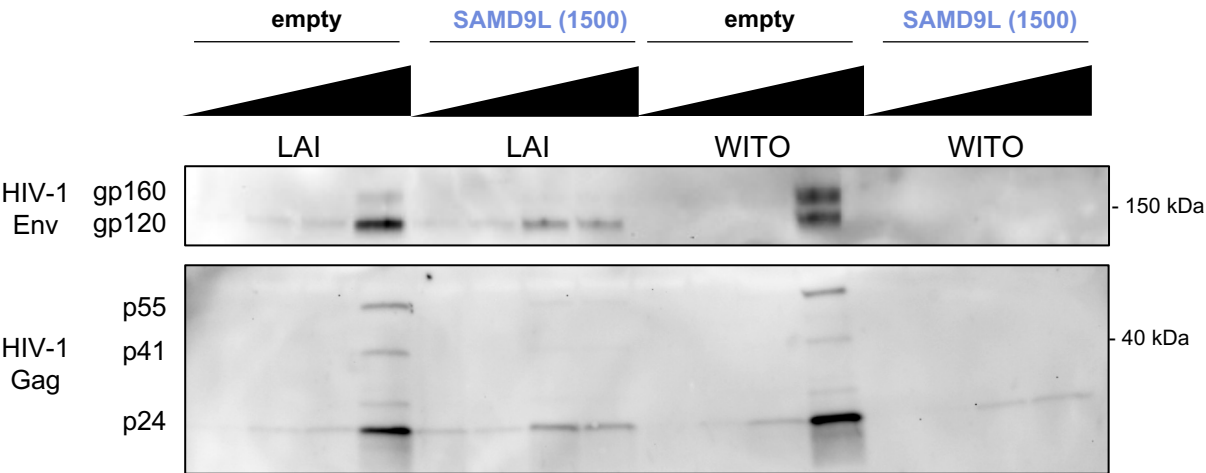

Figure S4

Supplement: S4 Fig — Western blot analyses of HIV-1 Env and Gag proteins in the viral producer cells (A) and in the supernatant (B). Cells were cotranfected with empty plasmid (control) or 1,500 ng of SAMD9L plasmid and with 400, 800, 1,200, or 1,600 ng of IMC for pLAI and pWITO (only 800 and 1,200 ng pWITO conditions are shown in the cell lysates). Quantifications are presented below the corresponding lanes for Env and Gag p24 protein expression, normalized to the total proteins in the cell fraction, and expressed as fold difference compared to the condition in the absence of SAMD9/9L normalized to 1. The raw images underlying this Figure can be found in S1 Raw Images. HIV-1, human immunodeficiency virus type 1; IMC, infectious molecular clone; SAMD9/9L, sterile alpha motif domain-containing proteins 9 and 9-like. (PDF) [file pbio.3002696.s004.pdf]

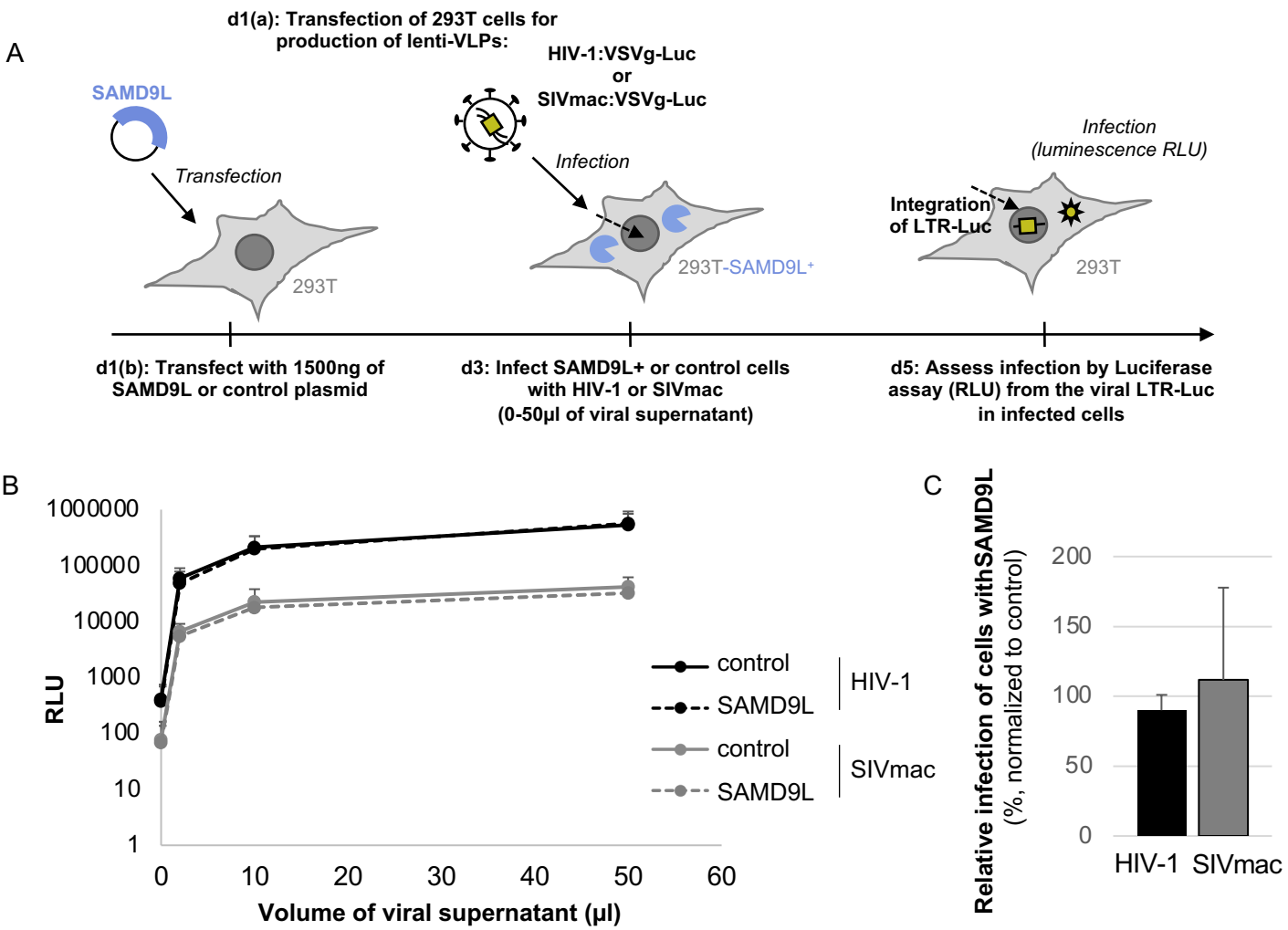

Figure S5

Supplement: S5 Fig — (A) Experimental setup (d1, day 1). Briefly, single-round lentiviral particles, HIV-1:VSVg-Luc and SIVmac:VSVg-Luc, were produced in 293T cells. Two days later, viral supernatant was collected and 1, 2, 10, or 50 μl was used to infect target cells overexpressing or not SAMD9L. Two days postinfection, cells were lysed and a luciferase assay was performed for titration. (B) Infectious virus yield to assess the impact of SAMD9L on the early phases of lentiviral replication. The Y-axis shows the RLUs in the luciferase assay. The data represent the mean of 3 independent experiments. (C) Corresponding relative infectious virus yield of HIV-1:Luc and SIVmac:Luc (from the 10 μl condition in B), normalized to the control condition (100%). RLU, relative luminescence unit; SAMD9L, sterile alpha motif domain-containing protein 9-like. (PDF) [file pbio.3002696.s005.pdf]

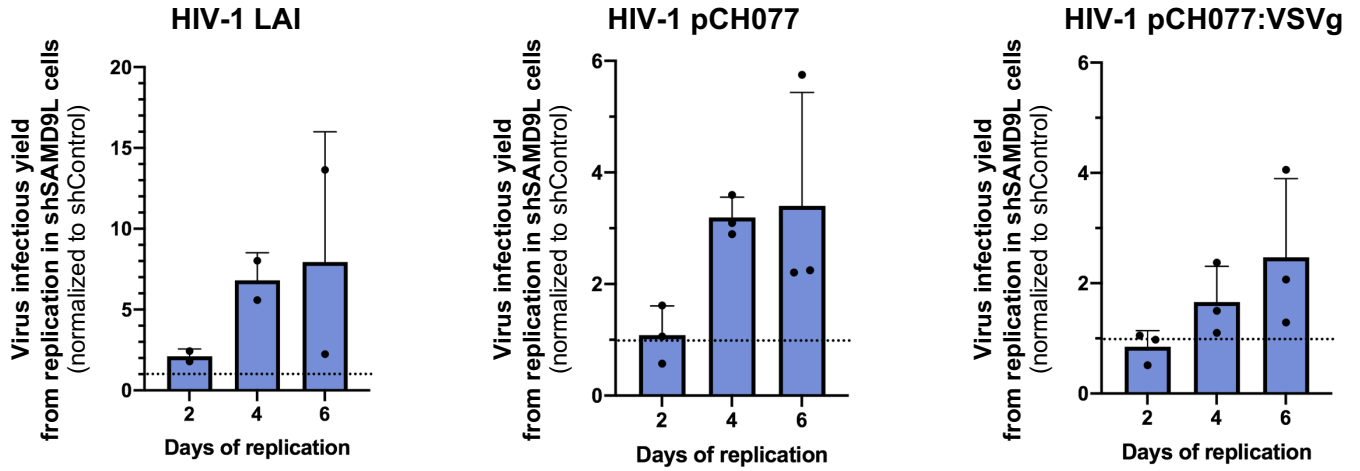

Supplement: S6 Fig — Experimental setup for the KD and viral replication experiments is shown in Fig 3B. Results from the replication experiments of HIV-1 LAI, HIV-1 T/F pCH077, and HIV-1 pCH077:VSVG in shControl or shSAMD9L HeLaP4P5 stimulated with IFNβ over 6 days of viral replication. Titration of the supernatant was performed using the TZM-bl assay at day 2, 4, and 6. Here are presented normalized means of the 3 independent biological replicates for HIV-1 pCH077 and pCH077:VSVg, and 2 for LAI (normalized to the titers in shControl conditions). The data underlying this Figure can be found in S1 Data. HIV-1, human immunodeficiency virus type 1; KD, knock-down; SAMD9L, sterile alpha motif domain-containing protein 9-like; T/F, transmitted/founder. (PDF) [file pbio.3002696.s006.pdf]

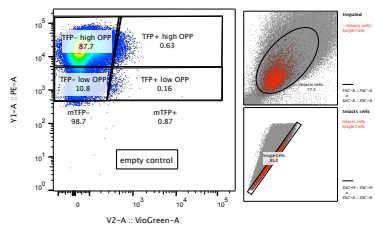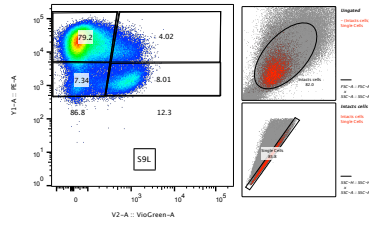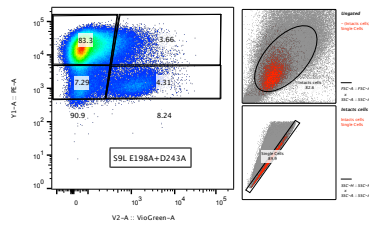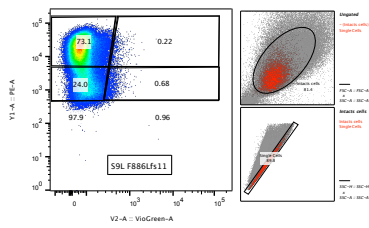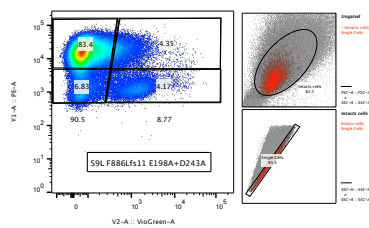

Supplement: S2 Data — (ZIP) [file pbio.3002696.s011.zip › S2_data/gating strategy.pdf]
