# Supplementary material for: SAMD9L acts as an antiviral factor against HIV-1 and primate lentiviruses by restricting viral and cellular translation
Source: PLoS Biol. 2024 Jul 3;22(7):e3002696. doi: 10.1371/journal.pbio.3002696 (PMC11221667; doi:10.1371/journal.pbio.3002696)
Supplement: S7 Fig — (A) SAMD9 crystal structure in complex with DNA (PDB 7KSP). Atoms of the residues forming the active site are represented with purple sticks (labeled in black), while those involved in DNA binding are in yellow. (B) Snapshot of the alignment of rSLFN13 and SAMD9L from HHpred analysis results. (C) Structural overlap of SLFN N-Domain including the SLFN-box of SLFN5, SLFN12, SLFN13, and SAMD9 AlbA2 domain. Residues forming the active site are indicated in purple below the schematic representation of SLFN family’s domains. (D) Representation of the SLFN-box, including the putative active site in purple, of SLFN5, SLFN12, SLFN13, and SAMD9. Coordinates are labeled in black. SAMD9, sterile alpha motif domain-containing protein 9; SAMD9L, sterile alpha motif domain-containing protein 9-like; SLFN, Schlafen. (PDF) [file pbio.3002696.s007.pdf]

A

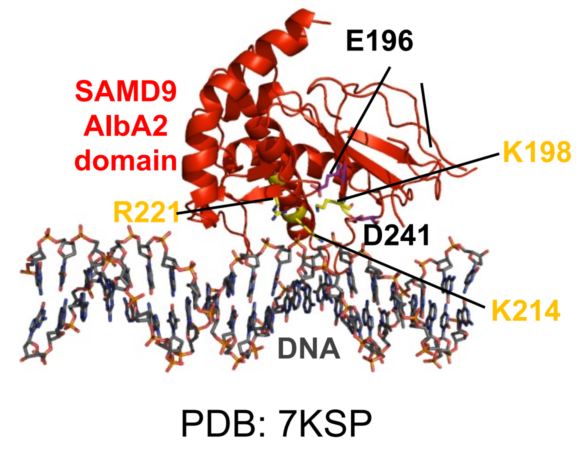

B

```
Probability: 87.22%,   E-value: 2.2,   Score: 45.66,   Aligned cols: 137,   Identities: 15%,   Similarity: 0.197,   Template Neff: 8.7

Q ss_pred              ccccccCCCCCcHHHHHHHhhhccchhhhhcCCCCCCeeeecccCCCCC-eeeceecC--CHHHHHHHHHHHHHHHcCH
Q Q_9189866    198     EFKALTNTETATEVDIKMKFSNEVFRFASACMNSRTNGTIHFGVKDKPHG-EIVGVKIT--SKAAFIDHFNVMIKKYFEE     274 (548)
Q Consensus    198     efk~~~~~~~~~~~~~~~~kf~~ev~rFa~acmN~rtNGTIhFGV~d~~~G-eIvGi~v~---kd~~~d~~~~~i~~~F~~     274 (548)
                       +||.+.....  ......++.+.+..||++      .-|+|+|||.|  .+ .++|+...  ..+.+.+.+...|.+....
T Consensus    223     EfK~~~~~~~~~~~~~~~~l~k~isAFANt-----~GG~l~iGV~d--~~~~~v~G~~~~~~~~~~~l~~~i~~~i~~~~~~     293 (366)
T 5YD0_B       223     EFKQFSTRRA--HEYIKSVIPEYISAFANT-----QGGYLLFGVDD--ESKRVLGCPKDNVDRDSLKAVVNEAISKLPVF     293 (366)
T ss_dssp              EEECCCSBCH--HHHHHHHHHHHHHHHHHHT-----TCEEEEESBCT--TTCBBCCEESTTCCHHHHHHHHHHHHHHTSCEE
T ss_pred              EEeecCcccH--HHHHHHHHHHHHHHHHhhC-----CCcEEEEEEeC--CCCEEeecCCCcCCHHHHHHHHHHHHHhCCCcc

Q ss_pred              HhHHHHHhccCCCCceEEEecCCCCCCcCeEEEEEEeeccccccCCCceEEEEecccCCcccccccccEEEEec-CCCccchh
Q Q_9189866    275     SEINEAKKCIREPRFVEVLLQNNTPSDRFVIEVDTIPKHSICNDKYFYIQMQICKDKIWKQNQNLSLFVRE-GASSRDIL     353 (548)
Q Consensus    275     ~~~~~a~~cir~P~Fv~V~~~~~~~~~~~~~VIEVDvvp~~~~~~~~~~~~f~v~~~~~~~~~~~~~~~~~~~~~~~f~R~-g~~s~~i~     353 (548)
                       ..............|+.|...+..  ..+|+.|.|.|....+-.                    .....|+|+ |.....+.
T Consensus    294     ~~~~~~~~~~~~~~i~~~~v~~~~~~~~~~~~~~VivI~V~~~~~~vf~-----------------~p~~y~~~~~~~~~~~~l~     353 (366)
T 5YD0_B       294     HFCSSKEKVSYKTRVIDVFKEGNL--YGYLCVIKVERFCCAVFS-----------------EAPISWMADKENGVYSLN     353 (366)
T ss_dssp              CSSSCCCCCEEEEEEEEESSSSE--EEEEEEEEECCCSSEEBS-----------------SSCBCEEEETTTEEEECC
T ss_pred              cccCCCCCceEEEEEEEeCCCCce--EEEEEEEEEcCceeeEee-----------------CCCceEEEeCCCcEEEeC

Q ss_pred              hcccccHHHHHHHHHH
Q Q_9189866    354     ANSKQRDVDFKAFLQN     369 (548)
Q Consensus    354     ~~~~~~~~~~~~~~~~~~~~~~~~     369 (548)
                       .      .++..++..
T Consensus    354     ~-------~e~~~~~~~~~~     363 (366)
T 5YD0_B       354     T------EKWVRMMVD     363 (366)
T ss_dssp              H------HHHHHHHHT
T ss_pred              H------HHHHHHHhh
```

C

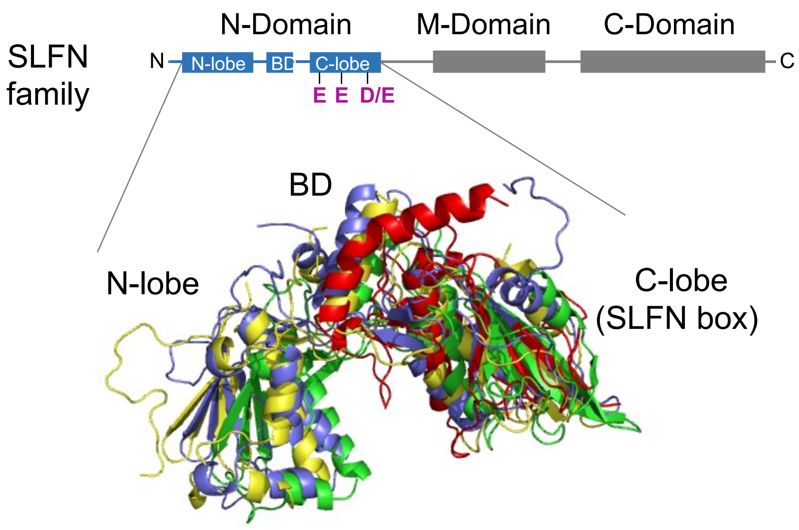

SAMD9 (156-385) PDB: 7KSP (Peng et al. 2021)
SLFN5 (1-336) PDB: 7Q3Z (Metzner et al. 2022)
SLFN12 (1-351) PDB: 7LRE (Garvie et al. 2021)
SLFN13 (2-341) PDB: 5YD0 (Yang et al. 2018)

D

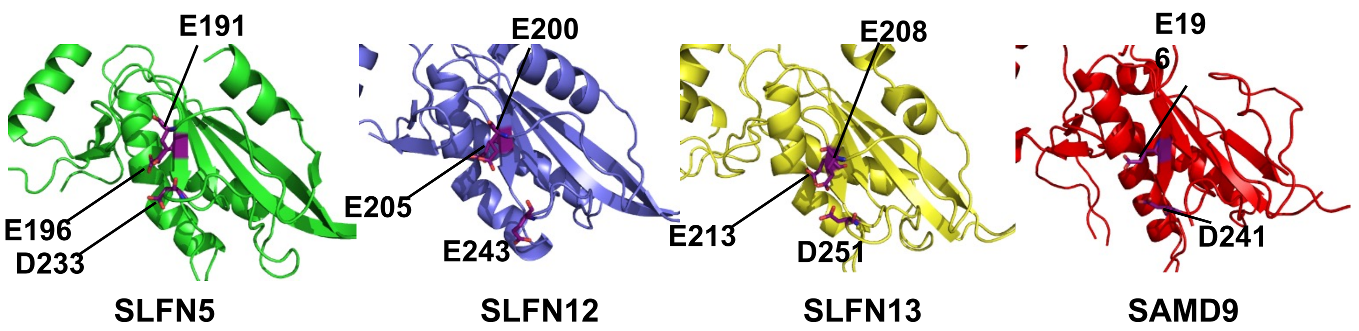

Figure S7
