# Supplementary material for: SAMD9L acts as an antiviral factor against HIV-1 and primate lentiviruses by restricting viral and cellular translation
Source: PLoS Biol. 2024 Jul 3;22(7):e3002696. doi: 10.1371/journal.pbio.3002696 (PMC11221667; doi:10.1371/journal.pbio.3002696)
Supplement: S1 Table — (DOCX) [file pbio.3002696.s008.docx]

**Table S1. Plasmids used in the study**

| **Name in the manuscript** | **Full name of the plasmid** | **Ref (acknowledgements and reference)** |
| --- | --- | --- |
| **HOST** | | |
| SAMD9L or TFP-SAMD9L | pMAX:mTFP1-SAMD9L | Tesi et al Blood 2017[1]; Shared by Yennan Bryceson |
| SAMD9L-E198A/D243A | pMAX:mTFP1-SAMD9L-E198A/D243A | This study |
| chSAMD9L | pcDNA3.1/V5-His-Topo:3xFLAG-chSAMD9L | Zhang et al JVI 2019[2]; Shared by Yan Xiang |
| chSAMD9L-D237A | pcDNA3.1/V5-His-Topo:3xFLAG-chSAMD9L-D237A | This study |
| SAMD9 | p3xFLAG-CMV19:hSAMD9 | Nagamachi et al Cancer Cell 2013[3]; Shared by Hirotaka Matsui |
| Empty | pcDNA3.1+ |  |
| FLAG-SAMD9L | pcDNA3.1/V5-His-Topo:3xFLAG-hSAMD9L | Zhang et al JVI 2019[2]; Shared by Yan Xiang |
| SAMD9L-F886Lfs*11 | pMAX:mTFP1-SAMD9L-F886Lfs*11 | This study |
| SAMD9L-F886Lfs*11-E198A/D243A | pMAX:mTFP1-SAMD9L-F886Lfs*11-E198A/D243A | This study |
| ISG20 |  | Wu et al plos Pathogens 2018[4] |
| **VIRUS: Replication competent IMCs**  The following reagents were obtained through the NIH HIV Reagent Program, Division of AIDS, NIAID, NIH: | | |
| HIV-1 LAI | pLAI | Shared by Michael Emerman |
| HIV-1 NL4.3 | pNL4-3 | Strain NL4-3 Infectious Molecular Clone (pNL4-3), ARP-2852, contributed by Dr. M. Martin |
| HIV-1 NLADA | Human Immunodeficiency Virus-1 ADA | ARP-416, contributed by Dr. Howard Gendelman |
| HIV-1 TF pWITO | Human Immunodeficiency Virus 1 pWITO.c/2474 | ARP-11739, contributed by Dr. John Kappes and Dr. Christina Ochsenbauer |
| HIV-1 TF pCH077 | Human Immunodeficiency Virus 1 pCH077.t/2627 | ARP-11742, contributed by Dr. John Kappes and Dr. Christina Ochsenbauer. |
| HIV-2 GL-AN | HIV-2 GL-AN | Kawamura et al 1994, PMID: 7898386 |
| SIVagm.Tan1 | SIVagmTan1 | Infectious Molecular Clone, ARP-3444, contributed by Drs. Marcelo Soares and Beatrice Hahn |
| SIVmac | SIVmac239 SpX ΔVprΔVpx | ARP-12250, contributed by Dr. Ronald C. Desrosiers. |
| **VIRUS: Virus-like particles** | | |
| VSVg | pMD2.G | Didier Trono Addgene 12259 |
| HIV-1 gagpol | pHIV-1 GagPol 8.2 | Didier Trono Addgene 12263 |
| HIV-1 LTR-GFP | pRRL-GFP | Didier Trono Addgene 12252 |
| HIV-1 LTR-Luc | pHIV-1-fLuc | Andrea Cimarelli |
| SIVmac gagpol | pSIV3+ | Nègre et al Gene Therapy 2000[5] |
| SIVmac LTR-Luc | pGAE-fLuc | Nègre et al Gene Therapy 2000[5] |
| SIVmac LTR-GFP | pGAE-eGFP | Nègre et al Gene Therapy 2000[5] |
| MLV gagpol | pTG5349 | Nègre et al Gene Therapy 2000[5] |
| MLV LTR-GFP | pTG13077 | Nègre et al Gene Therapy 2000[5] |
| psPAX2 | psPAX2 | Didier Trono Addgene 12260 |

**References**

1. Tesi B, Davidsson J, Voss M, Rahikkala E, Holmes TD, Chiang SCC, et al. Gain-of-function SAMD9L mutations cause a syndrome of cytopenia, immunodeficiency, MDS, and neurological symptoms. Blood. 2017;129: 2266–2279. doi:10.1182/blood-2016-10-743302

2. Zhang F, Meng X, Townsend MB, Satheshkumar PS, Xiang Y. Identification of CP77 as the Third Orthopoxvirus SAMD9 and SAMD9L Inhibitor with Unique Specificity for a Rodent SAMD9L. J Virol. 2019;93: e00225-19. doi:10.1128/JVI.00225-19

3. Nagamachi A, Matsui H, Asou H, Ozaki Y, Aki D, Kanai A, et al. Haploinsufficiency of SAMD9L, an endosome fusion facilitator, causes myeloid malignancies in mice mimicking human diseases with monosomy 7. Cancer Cell. 2013;24: 305–317. doi:10.1016/j.ccr.2013.08.011

4. Wu N, Nguyen X-N, Wang L, Appourchaux R, Zhang C, Panthu B, et al. The interferon stimulated gene 20 protein (ISG20) is an innate defense antiviral factor that discriminates self versus non-self translation. PLOS Pathogens. 2019;15: e1008093. doi:10.1371/journal.ppat.1008093

5. Nègre D, Mangeot PE, Duisit G, Blanchard S, Vidalain PO, Leissner P, et al. Characterization of novel safe lentiviral vectors derived from simian immunodeficiency virus (SIVmac251) that efficiently transduce mature human dendritic cells. Gene Ther. 2000;7: 1613–1623. doi:10.1038/sj.gt.3301292
