# Supplementary material for: SAMD9L acts as an antiviral factor against HIV-1 and primate lentiviruses by restricting viral and cellular translation
Source: PLoS Biol. 2024 Jul 3;22(7):e3002696. doi: 10.1371/journal.pbio.3002696 (PMC11221667; doi:10.1371/journal.pbio.3002696)
Supplement: S2 Table — (DOCX) [file pbio.3002696.s009.docx]

**Table S2. Primers for qPCR used in the study**

| **Primer name** | **Sequence** |
| --- | --- |
| U6 Forward | CGCTTCGGCAGCACATATAC |
| U6 Reverse | AAAATATGGAACGCTTCACGA |
| TBP Forward | CCCATGACTCCCATGACC |
| TBP Reverse | TTTACAACCAAGATTCACTGTGG |
| HIV-1 5’LTR Forward | CTTAAGCCTCAATAAAGCTTGCCT |
| HIV-1 5’LTR Reverse | CTAGAGATTTTCCACACTGACTAAAAGG |
| HIV-1 Gag Forward | GTAATACCCATGTTTTCAGCTTTATCAG |
| HIV-1 Gag Reverse | CATTCTGCAGCTTCCTCATTGAT |
