# Supplementary material for: SAMD9L acts as an antiviral factor against HIV-1 and primate lentiviruses by restricting viral and cellular translation
Source: PLoS Biol. 2024 Jul 3;22(7):e3002696. doi: 10.1371/journal.pbio.3002696 (PMC11221667; doi:10.1371/journal.pbio.3002696)
Supplement: S1 Raw Images — Uncropped membranes and Stain-Free BioRad gels for Figs 1C, 2C, 2D, 3C, 4A, 5F, 6C, 6D, 7A, 7B, S1A, S3B, S4A, and S4B. Corresponding cropped blots are circled in red. (PDF) [file pbio.3002696.s012.pdf]

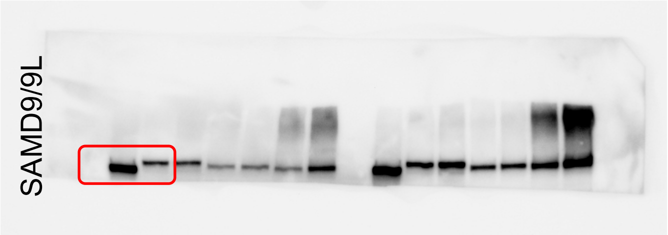

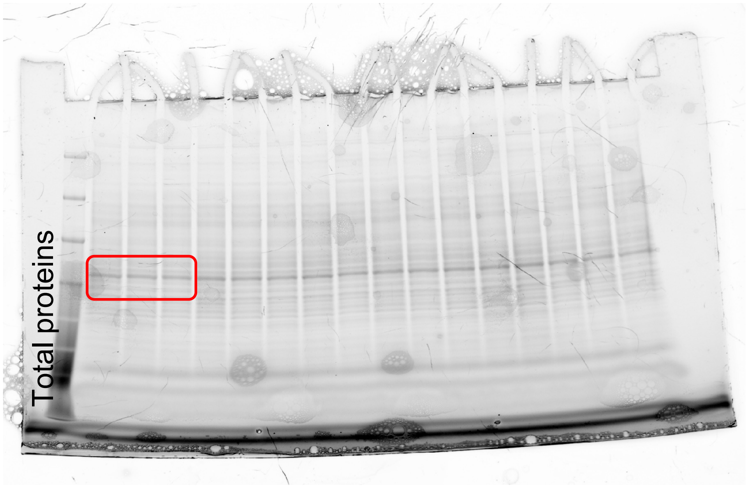

SAMD9/9L and Total proteins detection from Fig. 1C

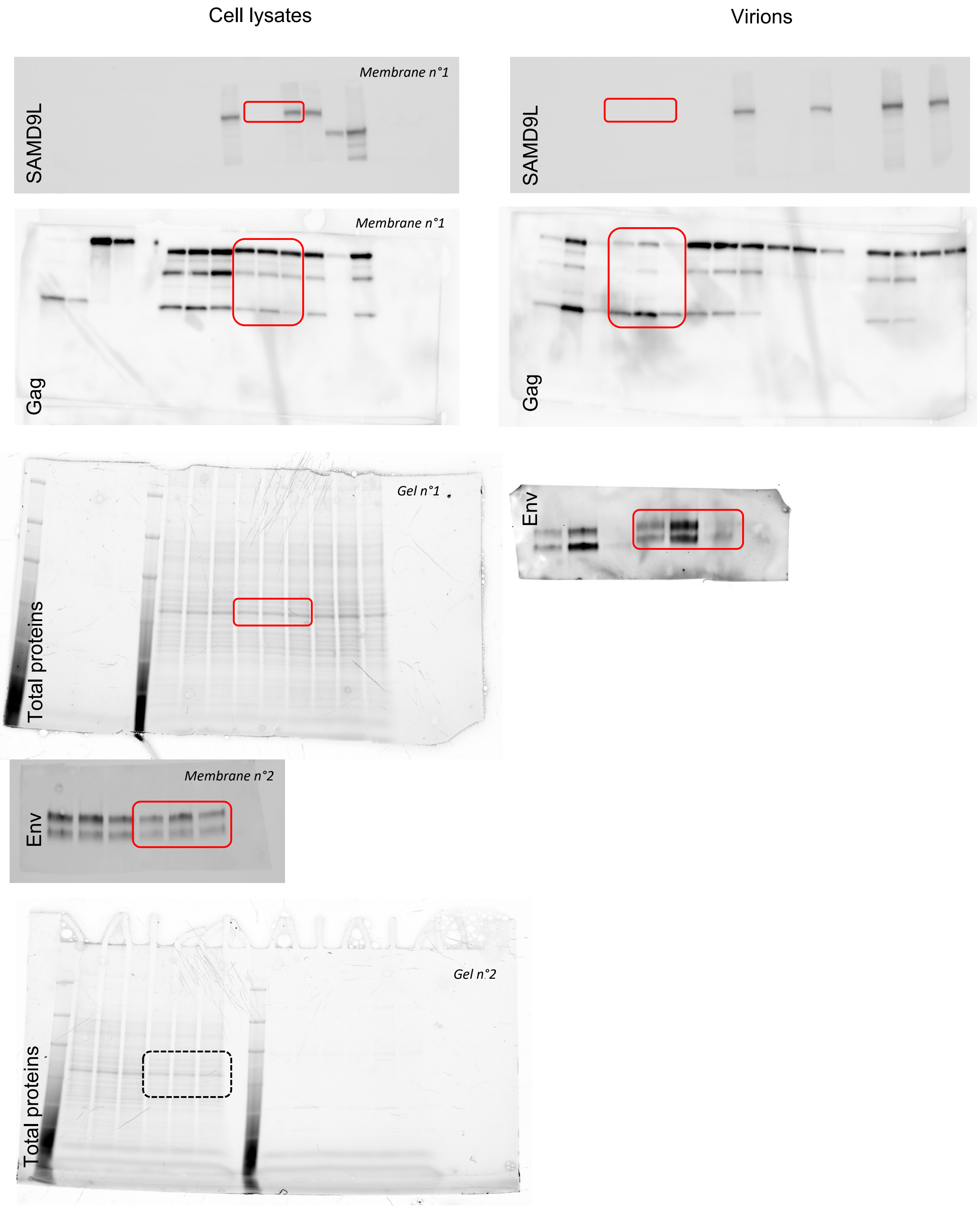

SAMD9L, Gag, Total proteins and Env detection from Fig. 2C (Left)

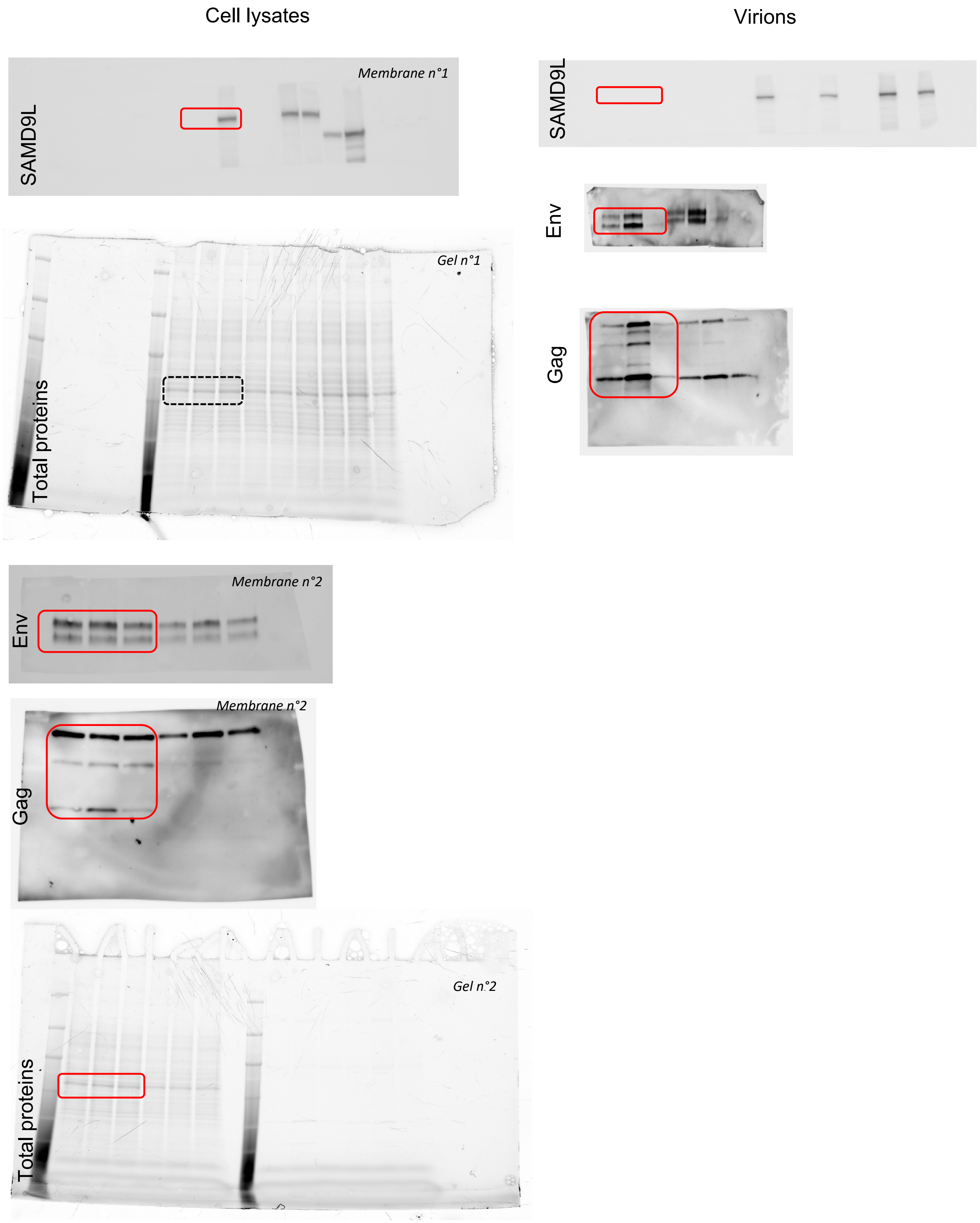

SAMD9L, Env, Gag and Total proteins detection from Fig. 2C (Right)

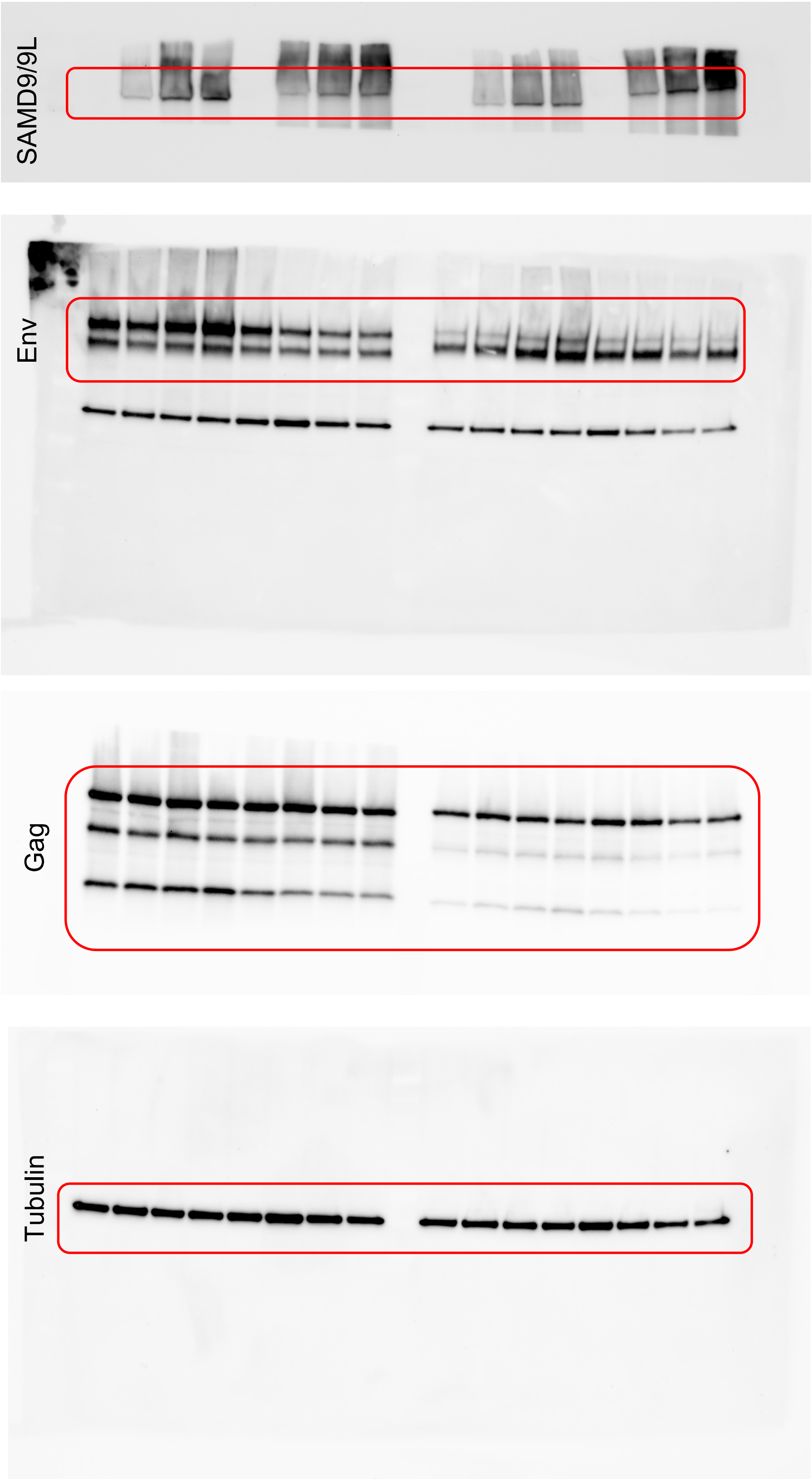

SAMD9/9L, Env, Gag and Tubulin detection from Fig. 2D

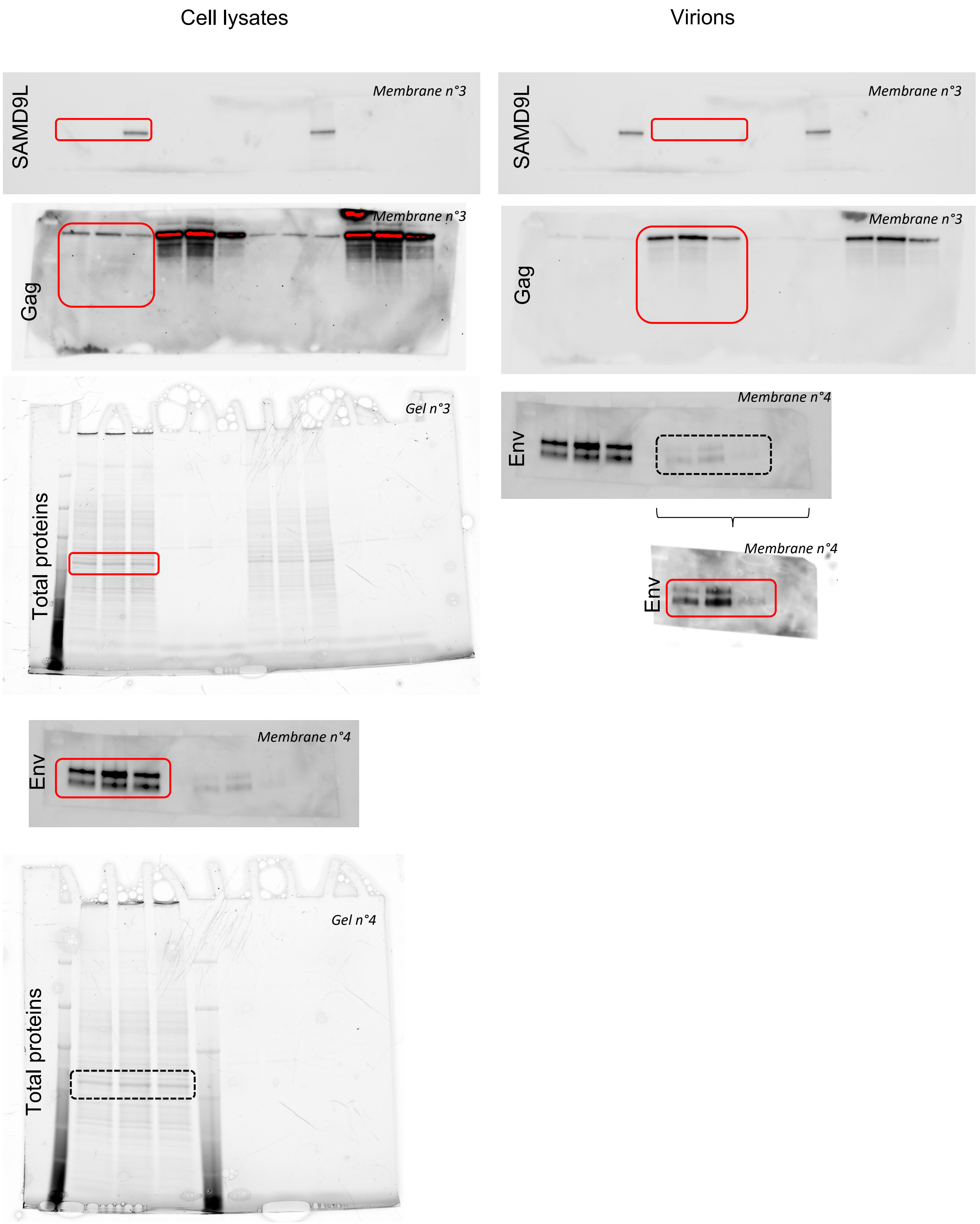

SAMD9L, Gag, Total proteins and Env detection from Fig. 3C

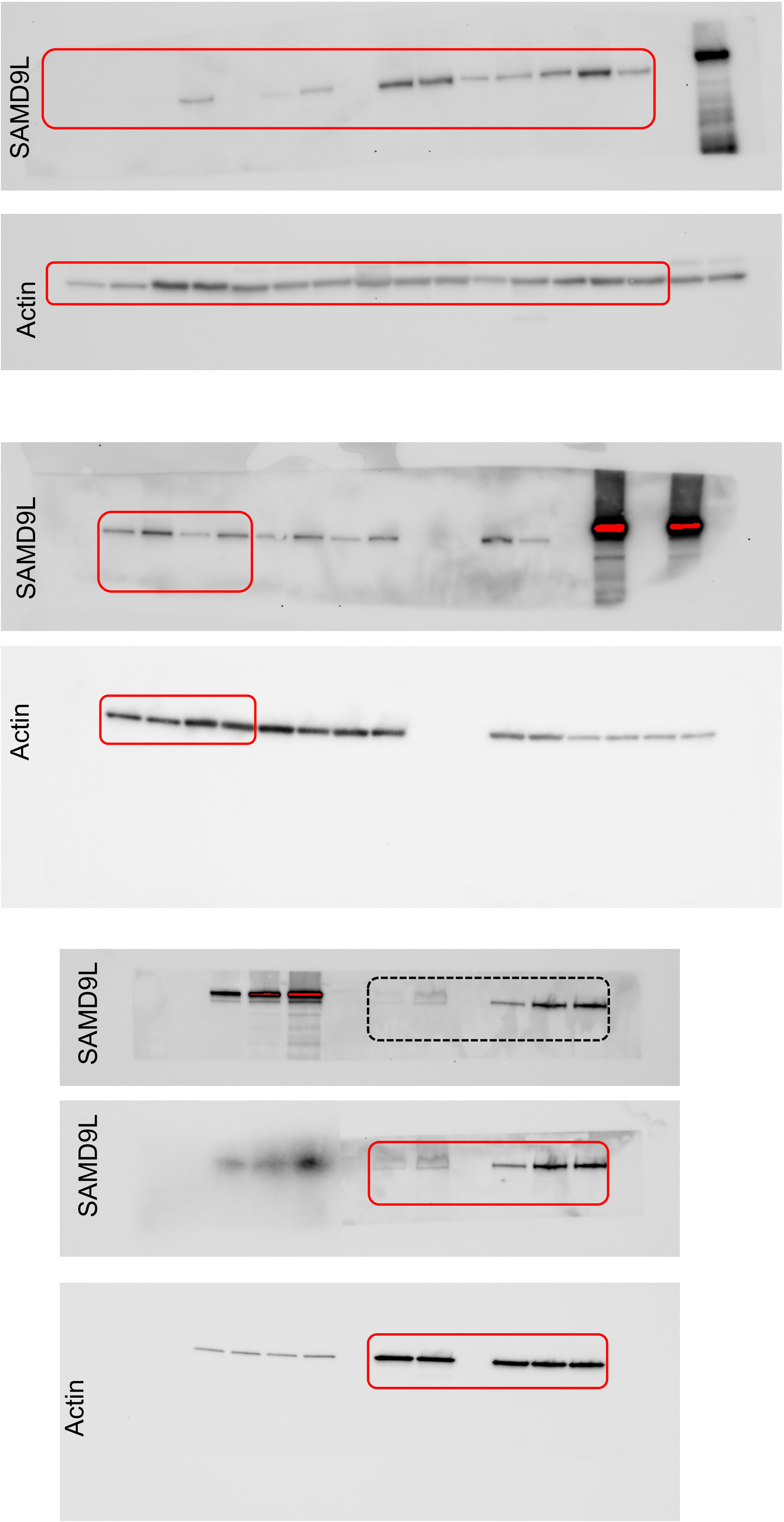

SAMD9L and Actin detection from Fig. 4A

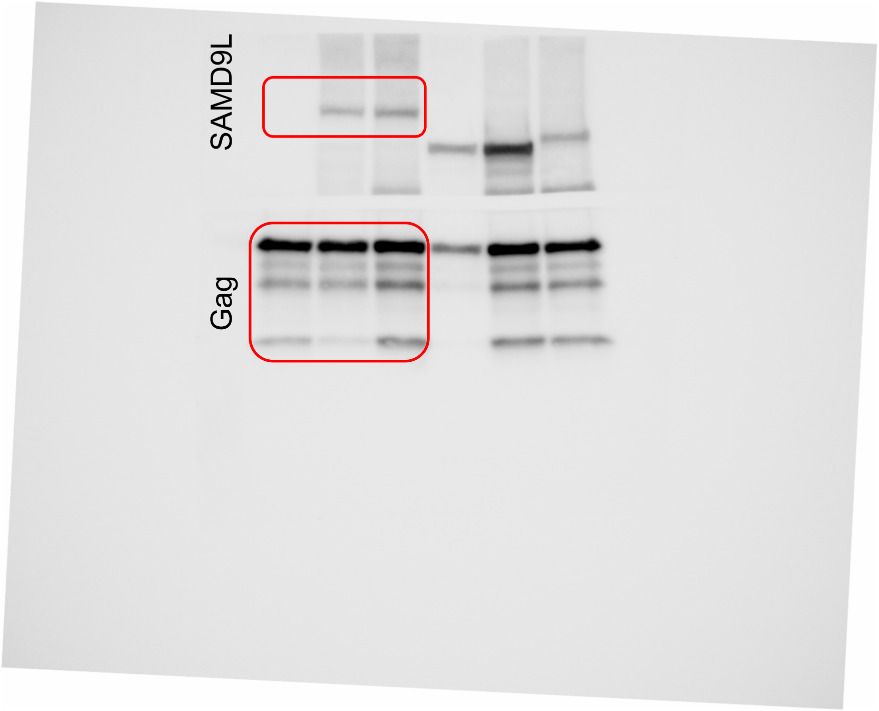

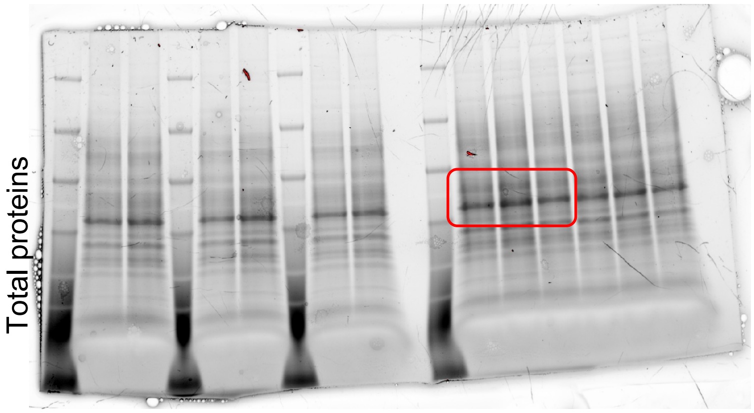

SAMD9L, Gag and Total proteins detection from Fig. 5F

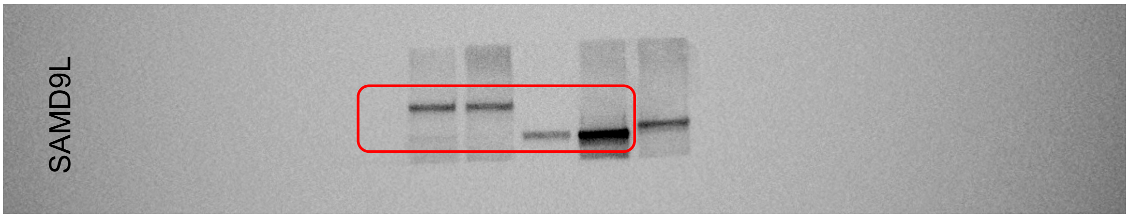

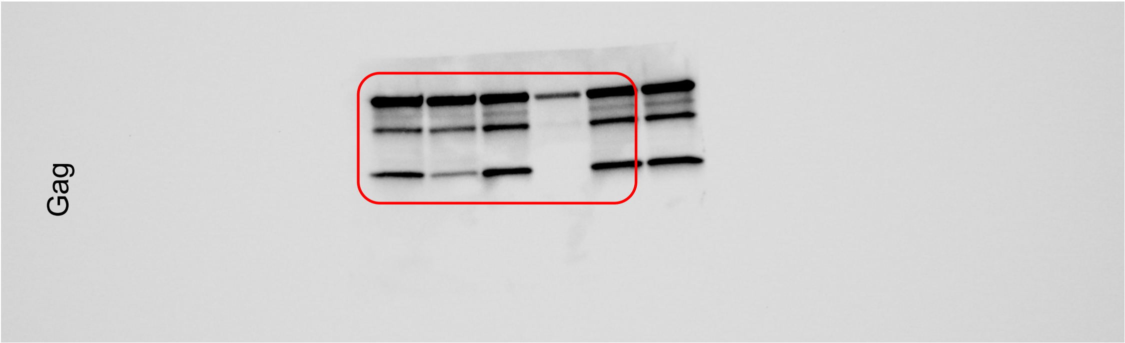

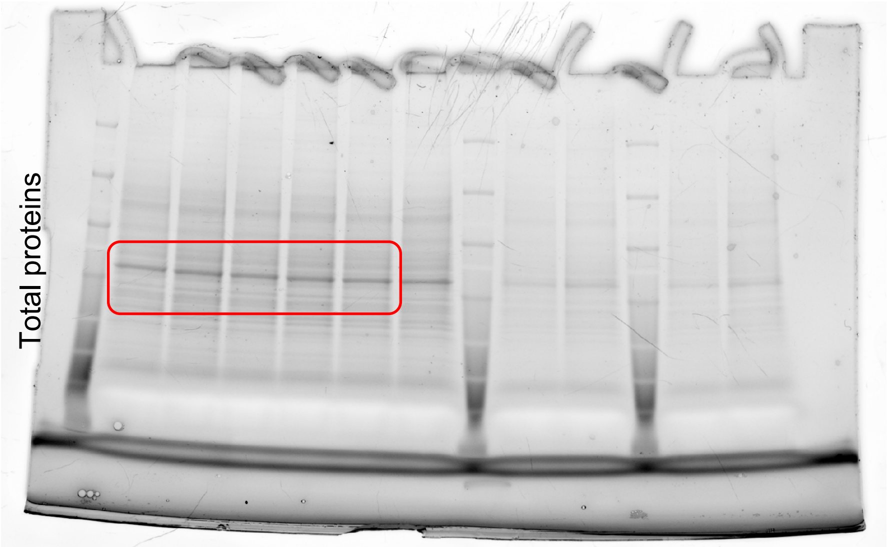

SAMD9L, Gag and Total proteins detection from Fig. 6D

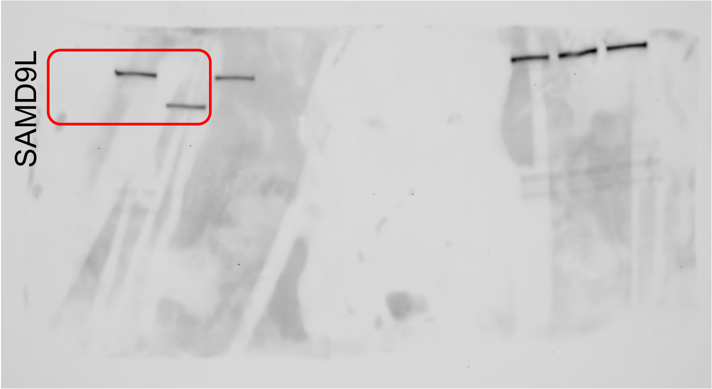

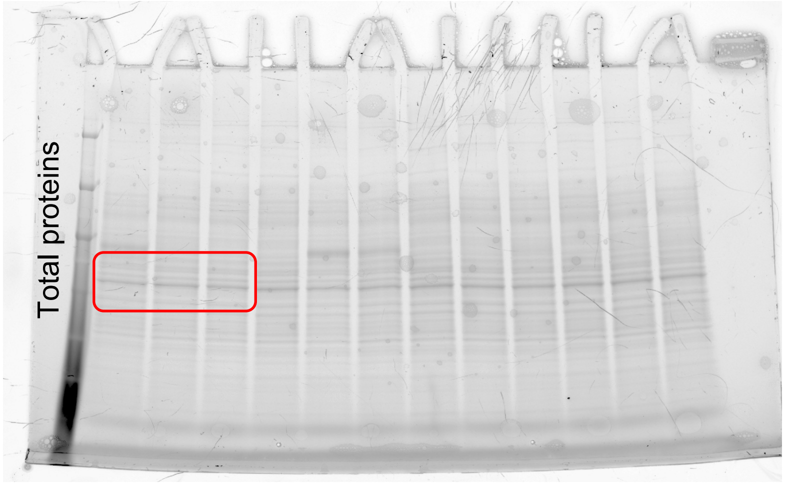

SAMD9L and Total proteins detection from Fig. 7A

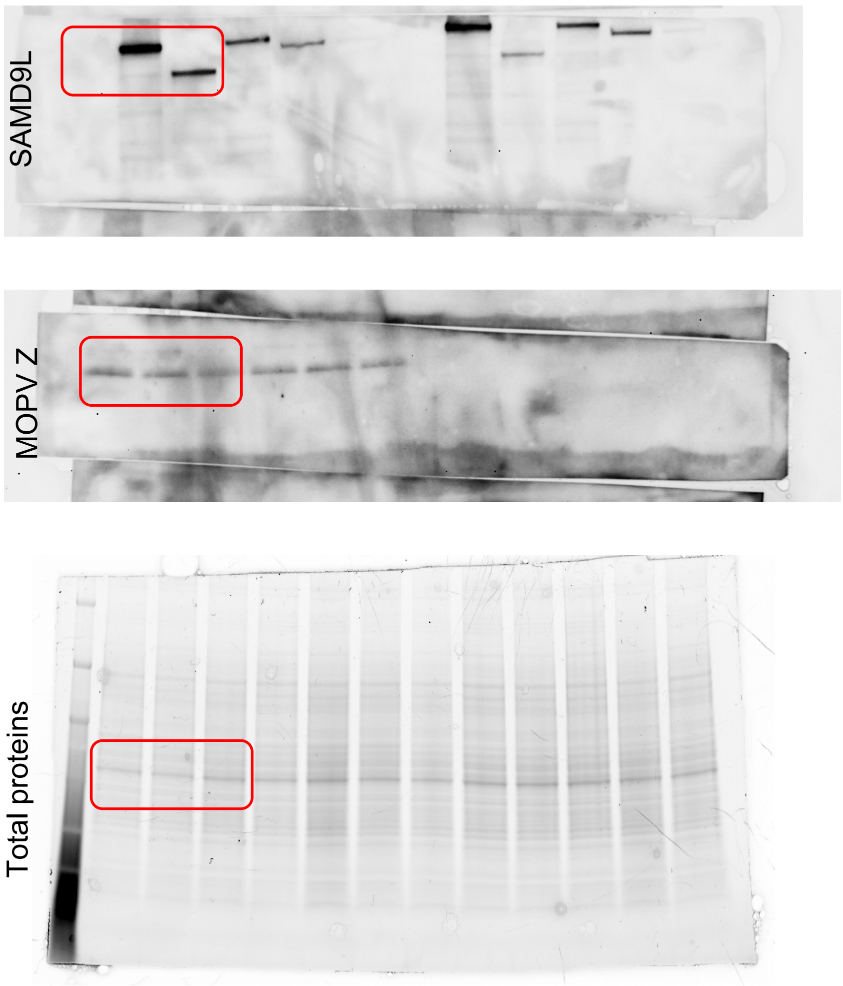

SAMD9L, MOPV Z and Total proteins detection from Fig. 7B

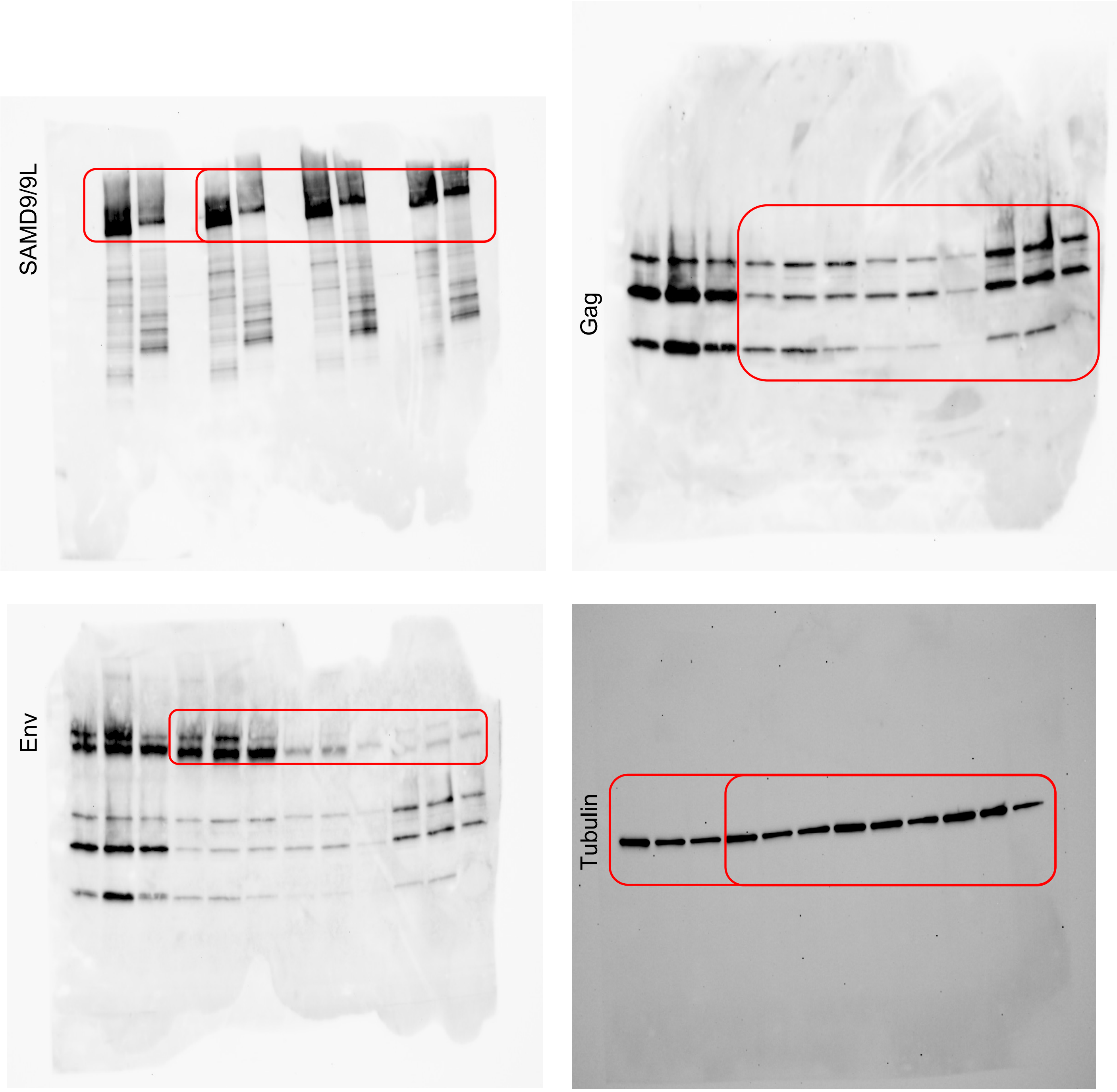

SAMD9/9L, Env, Gag and Tubulin detection from Fig. S1A (HIV-1) and S3B (HIV-1)

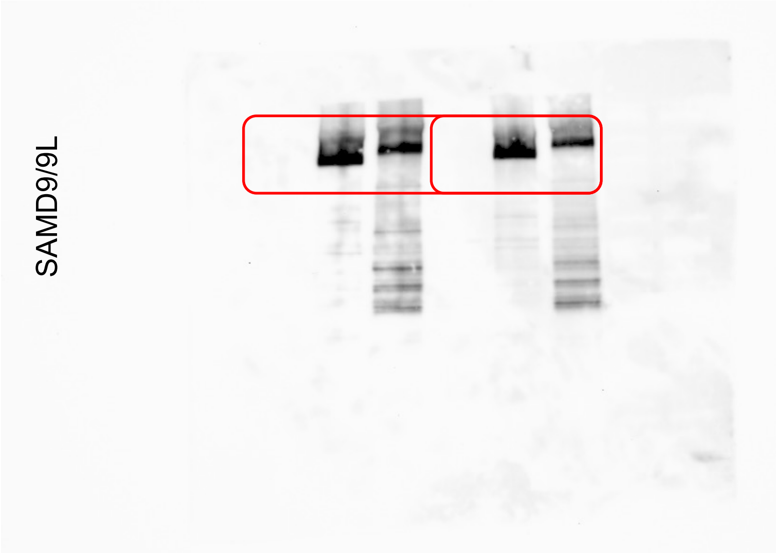

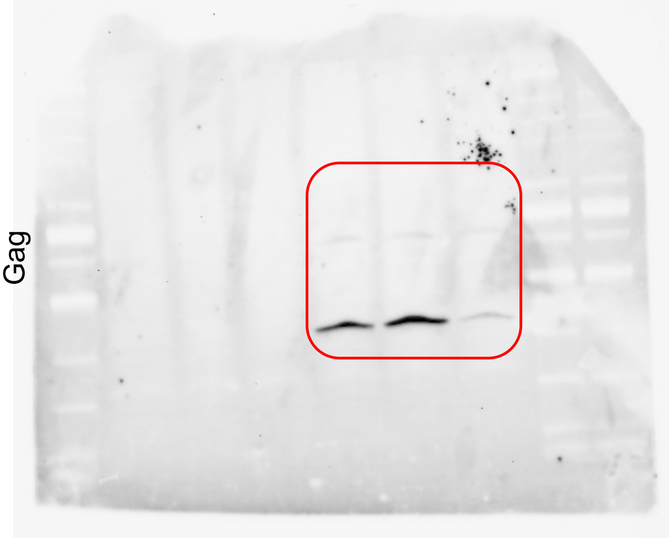

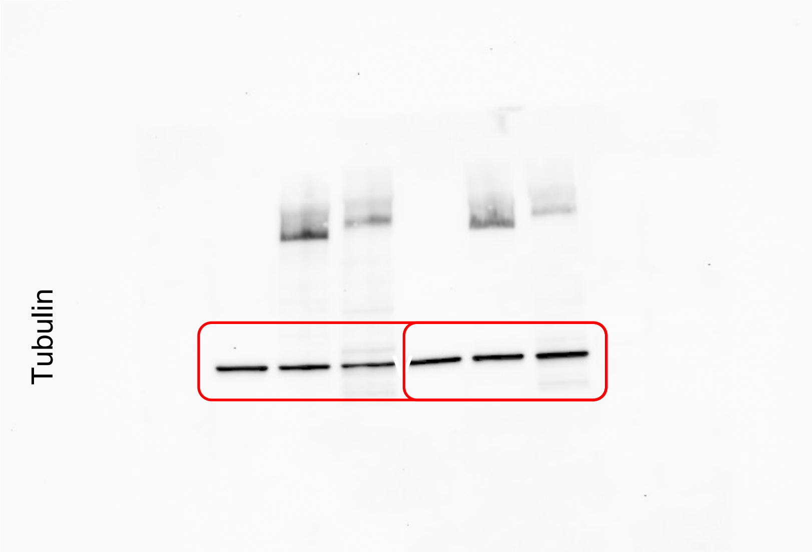

SAMD9/9L, Gag and Tubulin detection from Fig. S1A (SIV) and S3B (SIV)

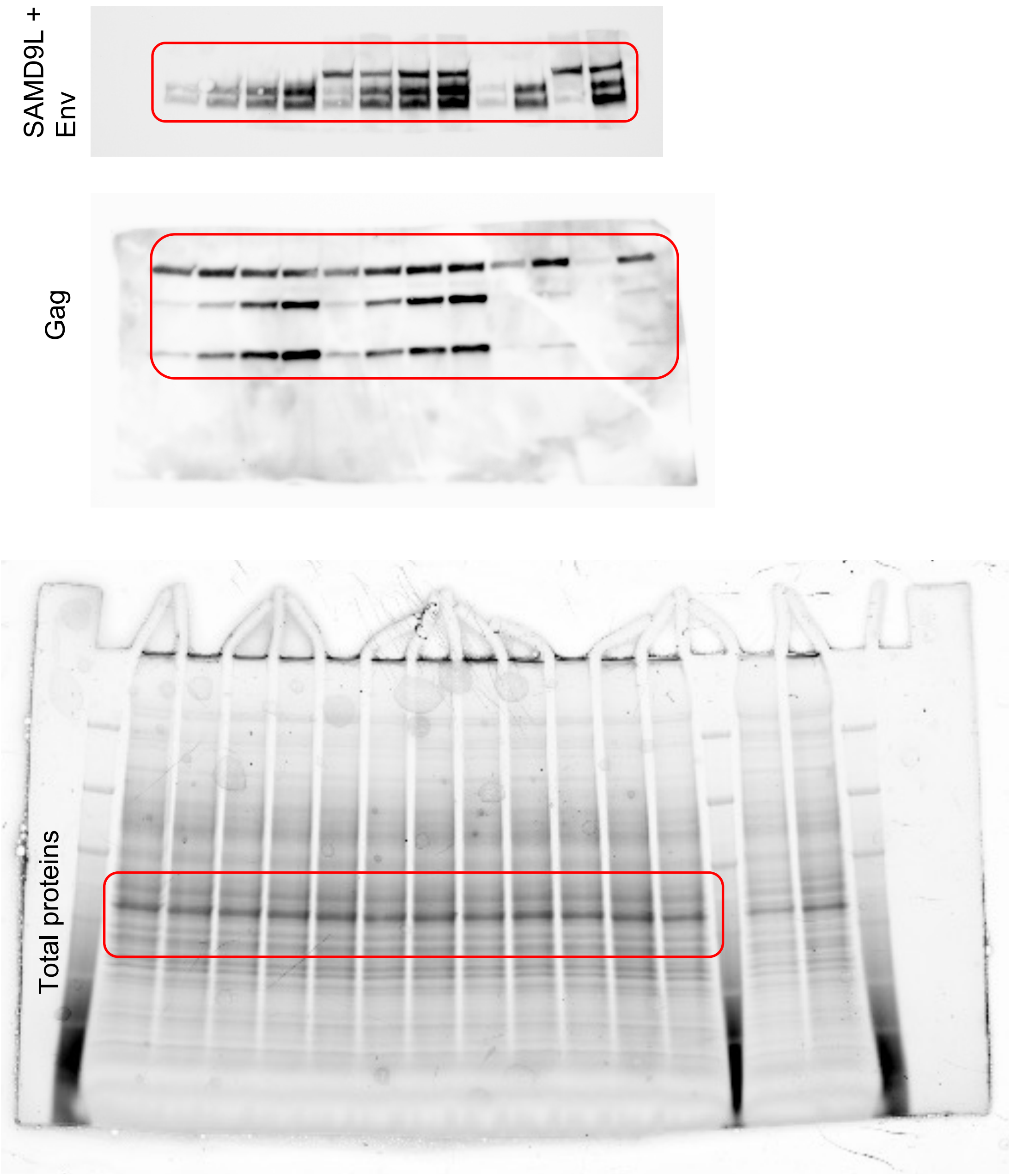

SAMD9L, Env, Gag and Total proteins detection from Fig. S4A

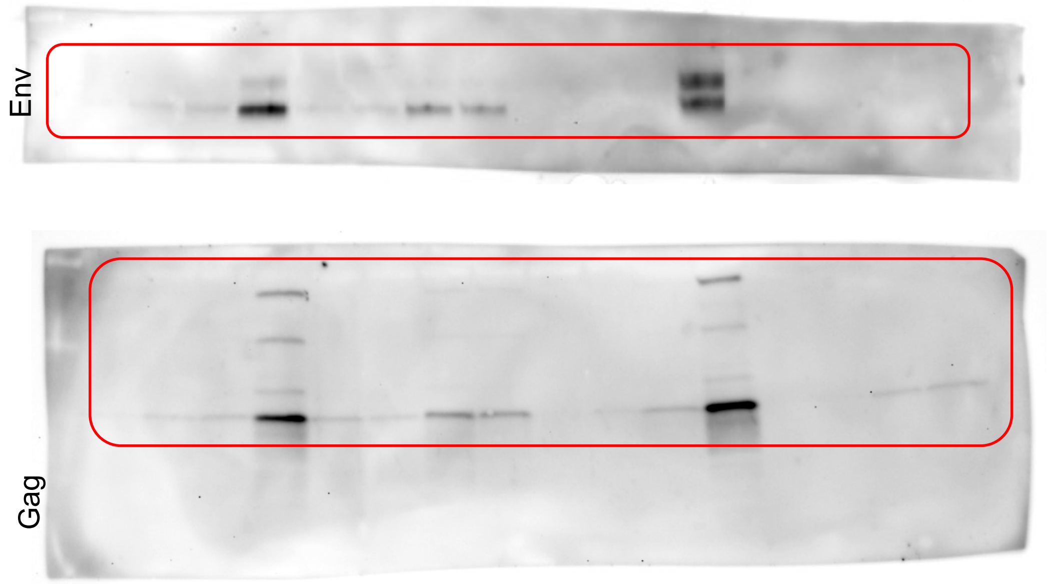

Env and Gag detection from Fig. S4B
